# Longitudinal national-level monitoring of on-farm broiler welfare identifies consistently poorly performing farms

**DOI:** 10.1038/s41598-021-91347-4

**Published:** 2021-06-07

**Authors:** Siobhan Mullan, Bobby Stuijfzand, Andrew Butterworth

**Affiliations:** Bristol Veterinary School, University of Bristol, Langford, Avon, Bristol, BS40 5DU UK

**Keywords:** Data processing, Systems analysis

## Abstract

A range of welfare outcome measures relating to on-farm welfare are monitored in UK slaughterhouses to check compliance with the European Broiler Directive. A national dataset from 438,155 batches of chickens between 2010 and 2014 and from 228,795 batches between 2016 and 2018 was analysed. The data contained information about 3.1 billion chickens. The highest mean proportion for a single condition was for ascites/oedema in 2016–2018 at 0.384%, affecting 3.9 million chickens/year sent to slaughter during that time, followed by abnormal colour/fevered at 0.324%, affecting 3.4 million chickens/year. Identifying farms most likely to have poor welfare is an important strategy for improving animal welfare overall, and for maximising the capacity for checking regulatory compliance when resources are limited. We found a greater proportion of broiler farms overall remained consistently in the best quartile (16.4%) rather than the worst quartile (6.6%). Farms that exceeded a Government ‘trigger’ threshold for poor welfare were significantly more likely to subsequently improve than ‘non-trigger’ farms, although they usually remained in the worst performing quartile of farms.

## Introduction

The European Broiler Directive^1^ concerning the welfare of chickens kept for meat production came into force in the UK on 30 June 2010. The Directive requires the collection and monitoring of on-farm mortality data and post-mortem condition data when the birds are slaughtered. This data is used to regulate stocking density above 33m^2^/kg up to a maximum of 42 kg/m^2^ (or 38 kg/m^2^ for the majority of farms in the UK, operating to the Red Tractor standard or equivalent) for farms that perform satisfactorily regarding bird welfare. The outcomes that must be monitored as specified in the directive are: daily mortality rate, cumulative daily mortality rate, number of birds dead on arrival at the slaughterhouse, as well as ‘other possible indications of poor welfare conditions such as abnormal levels of contact dermatitis, parasitism and systemic illness’. Across EU Member States the implementation of the directive in terms of the measures monitored and the process for ensuring sufficiently good welfare to allow a derogation above 33 kg/m^2^, is variable^2^.

In England and Wales, responsibility for monitoring broiler welfare through slaughterhouse-collected information lies with the Food Standards Agency (FSA). Every batch of chickens is monitored at the slaughterhouse, with information reported back to the FSA, who then alert the Governmental agency, the Animal and Plant Health Agency (APHA) when a batch of broiler chickens has exceeded a trigger threshold for any of the following conditions: ascites/oedema, cellulitis/dermatitis, dead on arrival, emaciation, joint lesions, respiratory problems, total rejections, cumulative daily mortality, or foot pad dermatitis score. These trigger thresholds for England and Wales are set at values that were (at the time when the thresholds were set in 2010) six standard deviations from the mean for a single measure, or when the cumulative daily mortality rate exceeded three standard deviations away from the mean, and three or more other measures exceed the mean^3^. The APHA and FSA have inspection regimes and data handling systems to communicate information relating to poor welfare between the slaughterhouse and the farmer. If triggers are exceeded, investigative action may be taken by APHA Veterinary Officers, on a risk basis, which can include requesting a written action plan and/or a visit to the production site^3^.

The data analysis presented here was undertaken as part of a larger project that developed a webtool to visualise both individual farm data, and to allow viewing of aggregated data for the welfare measures collected under the Broiler Directive. The webtool utilises data from England and Wales collected in slaughterhouses since 2010 and has the capacity to display data in relation to benchmarks, and over time. In addition, predictive models based on analysis of the data were developed as part of the webtool, to indicate to farmers the likely subsequent performance of their flocks, with the aim of encouraging mitigating action by the farmer if poor welfare was, or is, predicted. The data presented in this study includes an analysis of the risk factors for broiler welfare outcomes derived from ancillary data collected by the slaughterhouse. This approach is proposed as being ‘valuable’ by Averos et al.^4^ who analysed retrospective data from a single slaughterhouse in Spain. It is clear that the analyses that are possible are determined by the availability of data. Averos et al. had additional transport-related data and Buzdugan et al.^5^ present both retrospective and longitudinal data on risk factors for poor welfare from a single integrator in the UK. Previously Part et al.^6^ identified significant relationships with maximum daily temperature for ascites, abnormal colour and mortality during transport and lairage, hence we also analysed the associations between bird measures and meteorological data, and included both ancillary and meteorological data as the basis for the predictive models.

Government agencies have limited resources with which to conduct regulatory activities and targeting those farms most at risk of persistently poor welfare is likely to be important to optimise use of government agency time and effort, and to maximise welfare benefit. Our exploratory study analyses farm performance over time, and also assesses the performance of farms following breach of a trigger threshold, with the aim of increased understanding of the impact of regulatory activities, to help ensure the most effective use of welfare enforcement resources.

## Materials and methods

### Datasets

This study made use of data from two providers:

1. Data on broiler health and welfare derived from the FSA/APHA dataset collected in regard to the Broiler Directive (EC, 2007) was provided by Food Standards Agency (Operations Group) (FSA Ops) for two time periods: between 2010 and 2014 and between 2016 and 2018. Data in between these two periods was not available due to uncontrollable circumstances at the data supplier. Data from the two time periods was structured slightly differently and contained different variables, and as such will be treated separately throughout the rest of this report, unless explicitly stated.
  This data is collated in slaughterhouses by incorporating information about the number of birds arriving in each vehicle for slaughter (known as a ‘batch’) with the observations of trained meat inspectors who, after slaughter, tally up the number of birds in each batch affected by health conditions during the process of ensuring meat unfit for human consumption does not enter the human food chain. The data is then entered electronically in the slaughterhouse and sent to the FSA each day.
2. Weather data provided by the UK Met Office ranging from 2010 to 2018.

#### Broiler data 2010–2014

Data set 1: Broiler data was available from 30 June 2010–8 June 2014, and contained information from 438,155 batches of birds, where a batch is defined as a lorry load of birds arriving at the slaughterhouse.

During data screening we found that many batches contained unlikely or incorrectly recorded values on the ‘age’, ‘number of birds’, and ‘mortality in house’ variables. As such, using our own experience and in consultation with a UK broiler data expert and others in the field, we derived a number of selection criteria to determine whether observations could be included in the analysis or not. Only those batches from intensive indoor production aged between 20 and 60 days and between 20 and 120 days for other production types at the time of slaughter were retained for analysis. Houses recorded as containing either 0 or > 60,000 birds were treated as missing data. Finally, batches with recorded mortality in house < 0.5% or > 15% were dropped from the analysis.

After data cleaning, 296,986 batches were retained for inclusion in analysis. These batches represented data for 2,017,708,343 individual birds. We note that batches could have missing values for one or more variables, and as such the actual number of batches analysed varied between analyses, depending on which variables were included in the relevant analysis. The causes of missing data were unknown and varied and we could not assume that, for example, missing data was missing at random, missing not at random or missing completely at random and we considered that testing for randomness did not add anything to the analysis. Although we tried to retain as much data as possible for analysis, including by retaining all other data from a batch with one or more missing values, imputation of data was never employed as it was not perceived to add value to the analysis due to the large numbers of records retained.

#### Broiler data 2016–2018

Data set 2: Broiler data was available from 1 April 2016–28 February 2018, and contained information from 228,795 batches of birds. The data underwent a cleaning process similar to that of the 2010–2014 data.

After data cleaning, 150,782 batches were included for analysis. These batches represented data for 1,115,390,442 individual birds. As with the 2010–2014 data, the actual number of batches analysed varied between analyses, depending on which variables were included in the relevant analysis.

Both sets of data included the count, and percentage of birds observed, with a number health and welfare outcomes of interest. Further, both data sets also contained a number of variables containing contextual information on the circumstances under which the broilers were reared. The full list of variables is reported in Table 1.

**Table 1:** Variables available in broiler datasets.

Variable name	Additional Information	Intended use
Abattoir ID	Identifier of abattoir	Grouping variable
Farm CPH number	Identifier of farm	Grouping variable
Farm house ID	Identifier of farm house	Grouping variable
Region	Derived from postcode of farm^7^	Grouping variable
Date^a^	Date of inspection at slaughterhouse^x^	Predictor
Number of animals	Number of animals in batch sent to slaughter^x^	Predictor
Number in house^b^	Number of animals in house on the farm^x^	Predictor
Production system^c^	Pre-categorised^+^: (intensive indoor; extensive indoor, free-range, organic)^y^	Predictor
Stocking density	Density of animals in kg/m^2^ (pre-categorised^+^: < 33 kg/m^2^, 33–39 kg/m^2^, > 39 kg/m^2^)^z^	Predictor
Breed	Breed of animals (pre-categorised^+^: Cobb, Hubbard, Hybro, Ross) ^y^	Predictor
Age	Age of birds in days^x^	Predictor
Mortality in house	Cumulative daily mortality rate in house^x^	Predictor
FPD Swedish score	Swedish score of footpad dermatitis^8^	Outcome
Abnormal colour fevered^b^	Count and percentage	Outcome
Antemortem rejects^b^	Count and percentage	Outcome
Ascites and oedema	Count and percentage	Outcome
Bruising and fractures^b^	Count and percentage	Outcome
Cellulitis^b^	Count and percentage	Outcome
Dermatitis and cellulitis^d^	Count and percentage	Outcome
Dermititis^b^	Count and percentage	Outcome
Dead on arrival	Count and percentage	Outcome
Emaciation	Count and percentage	Outcome
Hepatitis^b^	Count and percentage	Outcome
Joint Lesions	Count and percentage	Outcome
Jaundice^b^	Count and percentage	Outcome
Pericarditis^b^	Count and percentage	Outcome
Perihepatitis and peritonitis^b^	Count and percentage	Outcome
Respiratory disease^d^	Count and percentage	Outcome
Salpingitis^b^	Count and percentage	Outcome
Total rejections^d^	Count and percentage	Outcome
Tumours and nodules^b^	Count and percentage	Outcome

^a^Variable was used to compute the relevant time of year predictors in the statistical models.

^b^Variable was not available for 2010–2014 data.

^c^This variable has no variance for 2010–2014 data, as all recorded batches came from intensive indoor farming.

^d^Variable was not available for 2016–2018 data.

^x^Continuous data.

^y^Categorical data.

^z^Ordinal data.

^+^Pre-categorised data was supplied with the categories already determined.

#### Weather data

The weather data contained locality specific information on daily minimum and maximum temperatures, as well as the relative humidity recorded daily at 6:00 and 15:00. Temperature data was available for the full time period of the broiler data; however, relative humidity data was only available from 16 March 2011.

Temperature data was provided by the UK Government Meteorological Office (Met Office) using Ordnance Survey National Grid Map references. The data was linked to the broiler data by finding, for each farm, the centre point of the postcode area associated with this farm. The centre point was expressed as the average latitude and longitude coordinate of the farm region. These coordinates were then matched to the closest coordinates available in the weather data, and the temperature measurement associated with these coordinates was taken as the temperature maxima and minima for that farm on a given day.

Relative humidity was provided by the Met Office using site ID’s, where each of the 5000 site IDs had an associated postcode, noting that one site ID can contain multiple postcodes. The relative humidity data was linked to the broiler data by averaging, for each given timepoint, the relative humidity of all sector ID’s whose postcode fell within the postcode area of a given farm. This approach differed from the approach for the temperature data, as for the temperature data it was possible to determine the centre of a geographical region. For the relative humidity we did not have information available on where in a given postcode a sector ID was located and as such a centre point could not be determined.

### Data analysis

The data was analysed using R vs 3.6 with Tidyverse for data processing and R vs 3.6 LME4 vs 1.1 for the analysis. To investigate the association of risk factors, weather patterns, and other contextual variables, (from now on: we will refer to these as ‘predictors’) with outcomes, we aimed to build a comprehensive statistical model for each outcome separately, where the independent contributions of each available predictor on the relevant outcome could be examined. In order to derive this statistical model, several intermediate steps were taken, which are described below. We first noted that for all outcomes, we used the percentage outcomes instead of count outcomes to eliminate the effect of different batch sizes. The same distribution was used for all models and we assumed the percentages are continuous variables with normally distributed residuals. There were low levels of collinearity (*r* < 0.8) between predictor variables except for minimum and maximum daily temperature (*r* = 0.95). For all analyses we used the same method for statistical inference. The confidence interval does not include 0 and we have at times reported these to make interpretation easier, at other times we have included the p value.

#### Modelling of weather data

We first established how weather could be included as a predictor in the statistical models. As weather may affect the welfare and health of a bird over the course of its life^6^, by influencing housing conditions, it was not immediately clear at what timepoint in a bird’s life weather should be taken to act as a predictor of the outcomes. To find the optimal timepoint we assessed the impact of weather for the following time points:

- weather taken on the day of slaughter;
- weather taken 10 days before slaughter;
- weather taken 15 days before slaughter;
- weather taken 30 days before slaughter;
- average weather over the last 10 days of life;
- average weather over the last 15 days of life;
- average weather over the last 30 days of life.

For each of these weather variables, we fitted a new, separate regression models for each outcome on the weather variable (133 models), and we then compared the explained variance (expressed in R^2^) in the outcome between the different weather variables. The weather variable with the highest R^2^ in an outcome was selected as a predictor in the final model for that outcome. We included minimum temperature in the models, but the high collinearity between minimum and maximum temperature means the results could likely be interpreted as the effect of any measure of daily temperature.

#### Effect of time of year on weather data

The time of year is expected to influence the values of many of the broiler outcomes. The question is to what resolution time of year should be modelled, i.e. does including each month yield a more applicable statistical model than including each season? As time of year is not continuous, each different time point needed to be included in the model as a dummy variable. It was therefore useful to select the largest time period, hence producing the smallest number of dummy variables. For this reason, we only examined month and season, and did not look at finer scale weekly or daily level changes. Similar to the weather data, we fitted a regression for each outcome based on month, as well as on season, and we selected the predictor with the highest outcome R^2^.

#### Nested structure of the data

Each batch of broilers (effectively a lorry load) cannot be considered as an independent observation. Each batch is nested within (derived from) a farm house, a farm, and a region. In addition, batches were also considered as being nested within abattoirs. The implication of this nested structure was that observations that came from the same grouping variable, e.g. two batches from the same farm, would share some variance that would not be observed between batches coming from two different farms. This shared variance needed to be factored into the statistical model for any inferences made on the significance of relationships between predictors and outcomes to be valid. To establish which of these grouping variables had considerable impact on the outcomes, we fitted so called “variance components models”, where the variance observed for an outcome is decomposed by grouping variables^9^, revealing the proportion of variance in an outcome that could be attributed to each grouping variable. We aimed for parsimoniousness in the models in order not to overfit them, therefore, only if a grouping variable accounted for a substantial part of the variance (i.e. with a variance partition coefficient > 5%) did we consider it substantial enough to be accounted for in subsequent analyses.

#### Final models assessing the effect of predictors on outcomes

With the information on weather predictors, seasonality, and nesting structure available we proceeded with specifying for each outcome a statistical model including all predictors except Number of Animals which was only included for the outcome Dead on Arrival. If there was no substantial variance observed for any of the grouping variables, a multiple regression was used to investigate the associations of the outcome with the predictors. For any of the grouping variables which showed substantial variance, a multilevel model with the grouping variable included as a random effect on the intercept was created. Because the outcomes as well as the predictors differ between the 2010–2014 and 2016–2018 data, models were fitted separately for both time periods. The reference categories were the largest category, except for ‘month’ where January was used.

### Performance of farms over time

To ascertain how consistently farms performed over time, and in particular, whether there were farms that were consistently poor, we ordered farms into quartiles based on their performance in a time period of a given length. Performance here is defined as the mean percentage of birds with a given condition across all batches submitted by a farm in that time period. We repeated this approach for a number of consecutive time periods, so that we obtained a time-series of ordered farms.

#### Establishing an appropriate time window

To order farms into quartiles we first needed to obtain a percentile distribution of farms for each of the broiler conditions. This required creating a time interval;

- long enough that sufficient farms had sent off batches to the slaughterhouse during this period, noting that most birds are slaughtered at from 32 to 40 days, and so most farms have ‘new crops’ of chickens at approximately 7 week intervals, but also;
- short enough that we could repeat this time window of analysis a number of times to obtain a time-series to provide useful insight into changes in welfare measure outcomes.

To find an appropriate length of time for the ‘analysis window’, the cumulative count of farms that had sent off at least one batch of birds since the first day recorded in the dataset was plotted. The slope of the line decreased substantially between two and three months, and so a time-window of three months was chosen to be used.

There were conditions for which it was not possible, in every time window, to categorise farms into four different quartiles, for example; when more than 25% of farms had no birds with a specific condition. In these cases we categorised farms using the following logic:

- If, in a given time window, the relevant condition was not observed at all, all farms are categorised as being in quartile 1.
- If, in a given time window, the 25th percentile was still zero, but the 50th percentile was larger than zero, all farms below the 50th percentile were categorised as quartile 1, any farm between percentile 50–75 was categorised as quartile 3, and any farm above percentile 75 as quartile 4.
- Similarly, if only the 75th percentile was larger than zero, all farms below it were categorised as quartile 1, and farms above it as quartile 4.

#### The effect of breaching a trigger threshold on the subsequent condition of broilers

Trigger thresholds were identified by the Government agencies (Defra, FSA, APHA) for ascites/oedema, cellulitis/ dermatitis, dead on arrival, emaciation, joint lesions, respiratory problems, total rejections, cumulative daily mortality, or foot pad dermatitis score as described in Table 2.

**Table 2:** Trigger thresholds used by UK Government Agencies for detecting poor welfare performance of farms^3^.

Post-mortem condition	Process 2	Process 2 trigger level (%)
Ascites/oedema	2.02	0.21
Cellulitis and dermatitis	3.00	0.20
Dead on arrival	1.51	0.12
Emaciation	0.67	0.04
Joint lesions	0.43	0.02
Respiratory problems	9.28	0.49
Total rejections	11.76	1.11
Cumulative daily mortality	11.85	NA
FPD score*	167	60

Process 1. APHA will be alerted if the level of a post-mortem condition is exceptionally high (exceeds mean + 6SD).

Process 2. APHA will be alerted if the Cumulative daily mortality rate is unusually high (exceeds mean + 3SD = 7.37%) and, additionally, the rate of three or more post-mortem conditions is high (exceeds the mean).

*The FPD score is not a percentage but is a score of the severity of lesions (between 0 and 200) based on scoring 100 feet as either score 0, 1 or 2.

To see if breaching a trigger threshold by a farm affected the proportion of broilers with adverse conditions in subsequent batches, we looked at the values for the specific condition at the time of the trigger and compared this with that 10 weeks later. This 10-week delay should not be confused with the three-month window described above to obtain the percentile distribution across farms.

An example—For each farm, the average percentage of total rejections for all batches processed on the current date was calculated (i.e. we mention average here, as some farms may submit more than one batch at a certain date), and subtracted from the average percentage of rejections that was observed 10 weeks previously. If the relevant farm did not have any batches exactly 10 weeks previously, data from the next available date further in the past was used. The resulting change score represented the ‘change’ in percentage of a given condition between 10 weeks previously and the current date. In our analysis, larger change score values mean larger reductions, and are thus interpreted positively. For the same data from 10 weeks previously, we looked at whether triggers were issued for that condition. If there were multiple batches on the given date we used the highest value (e.g. if there were 2 batches with no triggers, and 1 batch with a trigger “2”, the 2 was used).

We applied this data to a multilevel regression, using the change score as an outcome, and the trigger data of 10 weeks previously as a categorical predictor. We accounted for within-farm variability by including a random intercept by farm.

The 10 week delay was selected as showing optimal results after comparing model performance (coefficient sizes of the predictors) for 6, 8, 10, and 12 week delays.

Finally, we investigated the effect of triggers on performance improvement of previously poorly performing farms.

To this end we used the quartile data, and specifically looked at whether the odds of a poorly performing farm moving from the fourth quartile in the preceding time window into a lower quartile in the current time window increased as a function of the triggers issued in the preceding time window. To analyse this, we ran a multilevel logistic regression, where the proportion of triggers received over all batches sent off in the previous time window predicted the likelihood of change. Within-farm variation is accounted for by including a random intercept by farm.

## Results

### Descriptive statistics on outcomes

#### Descriptive statistics

Table 3 shows the summary statistics for all outcomes of interest. The highest mean proportion for a single condition was for ascites/oedema in 2016–2018 at 0.384%, affecting 3.9 million chickens sent to slaughter, followed by abnormal colour/fevered at 0.324%, affecting 3.4 million chickens. The mean Swedish FPD (foot pad dermatitis score) was 71.9 and 25.1 out of a possible maximum of 200 for the period 2010–2014 and 2016–2018 respectively. Salpingitis and pericarditis were least frequently recorded at 0.001% and 0.016% in 2016–2018 respectively.

**Table 3:** Summary statistics on all outcomes.

Condition	Period	Median (%)	Mean (%)	SD	Min	Max	*n*	n Missing	Mean number of chickens affected per year*
Abnormal colour fevered	2016–2018	0.23	0.33	0.53	0.00	87.15	150,782	0	3,421,110
Antemortem rejects	2016–2018	0.00	0.06	0.30	0.00	31.67	150,782	0	622,020
Ascites oedema	2010–2014	0.14	0.29	0.44	0.00	33.00	296,986	0	2,534,020
	2016–2018	0.21	0.38	0.74	0.00	61.51	150,782	0	3,939,460
Bruising fractures	2016–2018	0.01	0.03	0.09	0.00	9.62	150,782	0	311,010
Cellulitis	2016–2018	0.12	0.21	0.35	0.00	23.68	150,782	0	2,177,070
Dermatitis	2016–2018	0.00	0.02	0.19	0.00	47.76	150,782	0	207,340
Dermatitis cellulitis	2010–2014	0.01	0.13	0.54	0.00	100.00	296,986	0	1,135,940
DOA	2010–2014	0.07	0.13	0.46	0.00	79.25	296,986	0	1,135,940
	2016–2018	0.08	0.13	0.41	0.00	81.58	150,782	0	1,347,710
Emaciation	2010–2014	0.00	0.04	0.13	0.00	25.63	296,986	0	349,520
	2016–2018	0.00	0.03	0.12	0.00	8.80	150,782	0	311,010
FPD Swedish**	2010–2014	37	70.10	66.70	0	200	21,302	275,684	n/a
	2016–2018	10	25.10	38.67	0	200	36,018	114,764	n/a
Hepatitis	2016–2018	0.02	0.05	0.20	0.00	54.55	150,782	0	518,350
Joint lesions	2010–2014	0.00	0.06	0.22	0.00	36.88	150,782	0	524,280
	2016–2018	0.00	0.01	0.08	0.00	16.04	296,986	0	103,670
Jaundice	2016–2018	0.00	0.05	0.17	0.00	15.00	150,782	0	518,350
Pericarditis	2016–2018	0.00	0.02	0.10	0.00	8.75	150,782	0	207,340
Perihepatitis/peritonitis	2016–2018	0.12	0.30	0.63	0.00	92.00	296,986	0	3,110,100
Respiratory	2010–2014	0.00	0.00	0.00	0.00	0.00	150,782	0	0
Salpingitis	2016–2018	0.00	0.00	0.04	0.00	10.00	150,782	0	0
Total rejections	2010–2014	1.00	1.19	1.17	0.00	96.00	296,986	0	10,398,220
Tumours/nodules	2016–2018	0.00	0.02	0.27	0.00	60.39	150,782	0	207,340

*Using number slaughtered in 2012 (873.8 m) or 2017 (1036.7 m) as appropriate^10^.

**The foot pad dermatitis (FPD) Swedish method score is not a percentage but is a score of the severity of lesions (between 0 and 200) based on scoring 100 feet as either score 0, 1 or 2.

#### Outcomes over time

We present the results of the daily mean outcomes over time, providing insights into trends over time, and also into seasonal patterns for the three most common outcomes, ascites, abnormal colour and FPD in Fig. 1. Other outcomes are presented in Supplementary Figs. S1–S16. Season has the most impact on ascites oedema, with higher levels over the winter months and lowest levels in summer. None of the outcome measures show a substantial improvement over time except FPD, however, following the data break it is not known whether FPD did improve across the industry or whether this is a result of a change in recording methods used.

**Figure 1:**
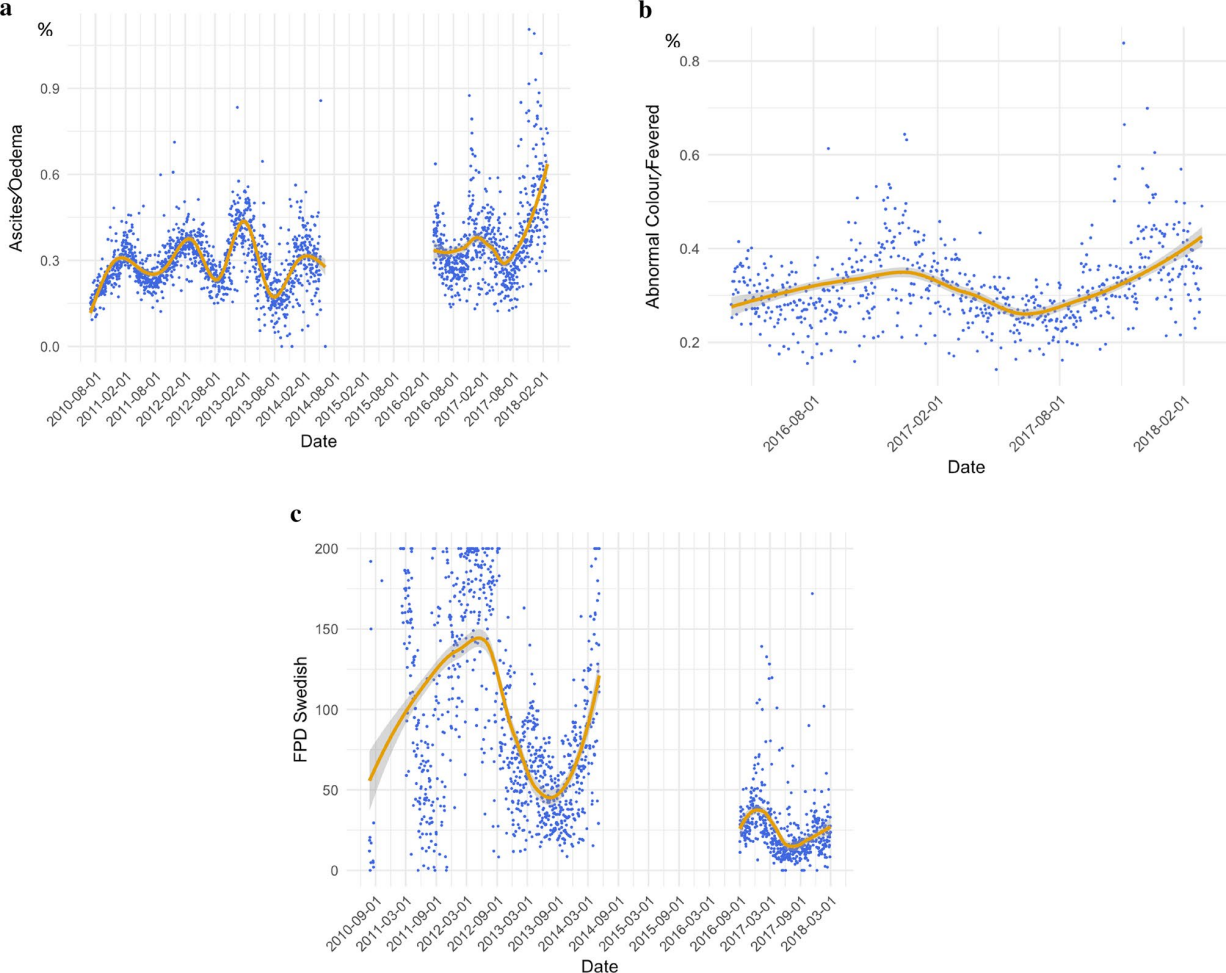
(**a**) Daily mean percentage of birds with ascites/oedema. (**b**) Daily mean percentage of birds with abnormal colour/fevered. (**c**) Daily mean foot pad dermatitis Swedish score. For all plots the daily mean value is represented by each dot. The line is the smoothing function to visualise trends (local polynomial regression fitting also known as LOESS) and associated shading denotes the 95% confidence interval.

### Predictors of outcomes

The following section details the results of the procedure described in the method section under ‘Data Analysis’: i.e. the various intermediate steps we undertook to arrive at the final statistical model for each outcome.

#### Appropriate modelling of weather data

To establish the best way of including weather into the statistical models, we compared the influence of different weather periods on each outcome (as expressed in explained variance: R^2^) and selected the period with the highest explained variance. Note that for this analysis we combined data for both 2010–2014 and 2016–2018 where possible. Supplementary Figure S17 shows the results by outcome, and for each type of weather. It reveals that for most outcomes the mean temperature or humidity over the last 30 days before slaughter yields the highest explained variance, hence this was used in all of the final models for consistency. For those outcomes, where other periods performed marginally better it was not considered substantial enough use an alternative period.

#### Effect of time of year on outcomes

We fitted regression models to establish if the time of year should be included in the statistical models by month, or by season. As with the weather data, this analysis combined data for 2010–2014 and 2016–2018 where possible. Supplementary Figure S18 shows the results and reveals that for each outcome, including month leads to more explained variance than season. As such, month was used as a predictor in the final models.

#### Nested structure of the data

For the last intermediate step we fitted variance component models to investigate how the nested structure of the data affected the variance observed in the outcomes. Again, for the purposes of this analysis data from 2010–2014 to 2016–2018 were combined where possible. Supplementary Figure S19 reports the proportion of variance observed in each outcome by grouping variable of Farm, House, Abattoir, Region. If the proportion of variance exceeded 5%, the grouping variable was included as a random effect in the final models.

### Final models assessing the effect of predictors on outcomes

#### Descriptive statistics on predictors

In Tables 4 and 5 summary statistics on the final set of predictors are reported.

**Table 4:** Summary statistics for continuous predictors.

	Period	Median	Mean	SD	Min	Max	n	n Missing
Age (days)	2010–2014	39	39.61	6.34	20	60	296,824	162
	2016–2018	37	38.46	8.10	20	120	150,782	0
Mortality house (%)	2010–2014	3.00	3.13	1.52	0.50	15.00	296,986	0
	2016–2018	3.59	3.93	1.74	0.50	15.00	150,782	0
N animals (in batch)	2010–2014	5544	6793.95	5434.16	27	96225^a^	296,986	0
	2016–2018	5588	7397.37	6230.82	6	177798^a^	150,782	0
N house	2016–2018	30,690	30,822.70	13,498.12	1	60,000	143,327	7455
Relative humidity 6 am (average 30 days)	2010–2014	91.83	91.44	2.95	77.47	100.00	221,264	75,722
	2016–2018	91.33	91.20	2.36	80.67	97.20	136,344	14,438
Relative humidity 3 pm (average 30 days)	2010–2014	72.17	72.35	8.96	46.03	100.00	221,264	75,722
	2016–2018	70.37	71.30	8.26	49.40	90.20	136,344	14,438
Temperature max (average 30 days)	2010–2014	13.40	13.45	5.52	− 1.62	26.53	295,993	993
	2016–2018	14.39	14.43	5.24	2.19	24.86	149,610	1172
Temperature min (average 30 days)	2010–2014	5.34	5.74	4.31	− 8.38	15.81	295,993	993
	2016–2018	5.85	6.61	4.15	− 1.91	15.79	149,610	1172

^a^Occasionally a number of batches were combined and reported in a single row hence the large maximum value.

**Table 5:** Summary statistics for categorical predictors.

Category	n 2010–2014	n 2016–2018
**Breed**		
Cobb	20,946	17,557
Hubbard	7000	5389
Hybro	1024	20
Ross	233,522	124,378
Missing	34,494	3438
**Stock density**		
< 33 kg/m^2^	61,346	18,659
> 39 kg/m^2^	3460	522
33-39 kg/m^2^	195,143	127,811
Missing	37,037	3791
**Production system**		
Extensive indoor		295
Free range		4739
Intensive indoor		138,996
Organic		2061
Missing		4691
**Month**		
1	26,405	14,163
2	23,567	12,286
3	26,088	6372
4	25,615	11,844
5	22,094	13,034
6	19,078	12,266
7	24,993	12,242
8	27,585	13,121
9	25,776	13,520
10	25,646	14,030
11	25,761	15,214
12	24,378	12,690

### Model results

**Figure 2:**
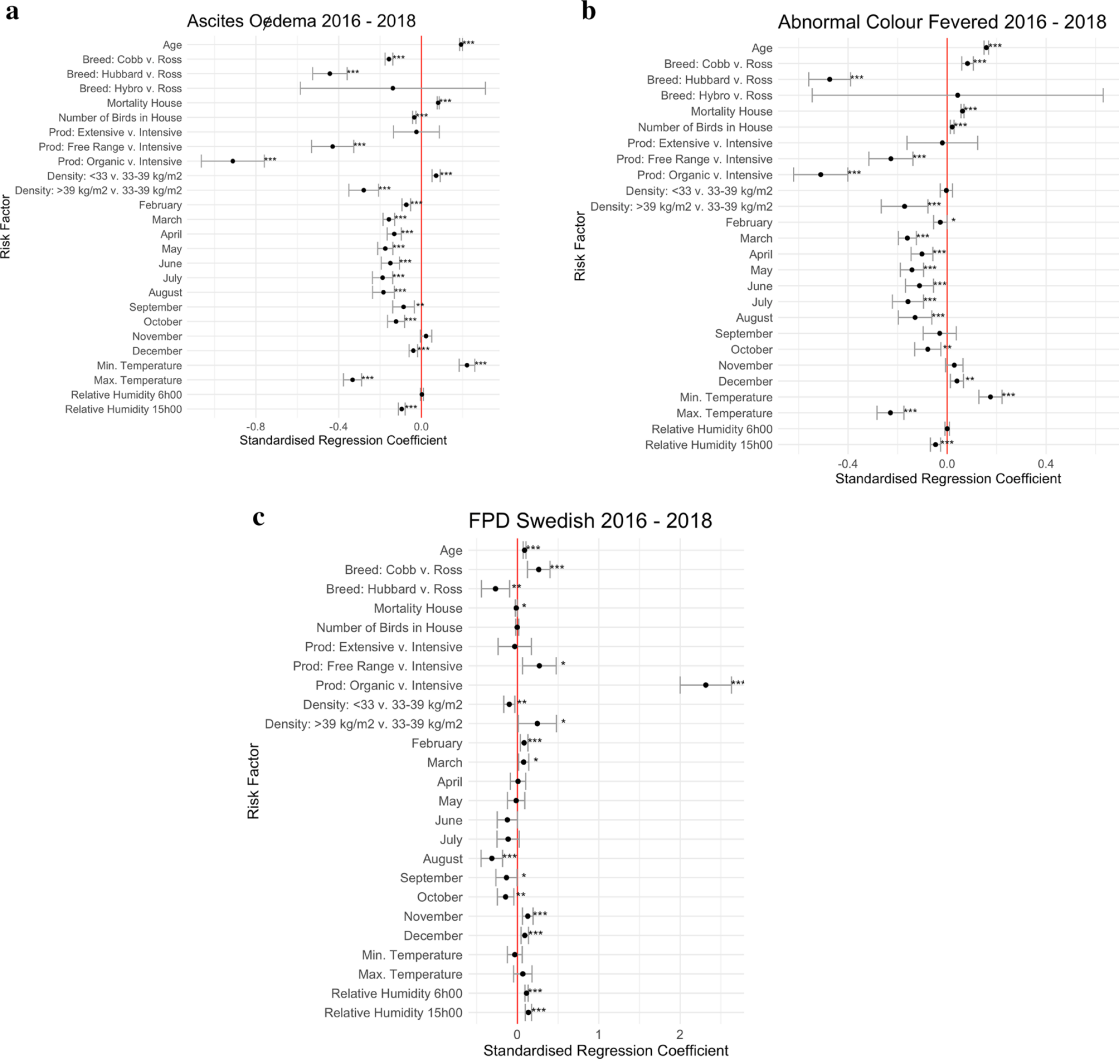
(**a**) Risk factors ascites/oedema in the 2016–2018 dataset. Confidence intervals are 95%. ***p < 0.001, **p < 0.01, *p < 0.05. (**b**) Risk factors for abnormal colour/fevered birds in the 2016–2018 dataset Confidence intervals are 95%. ***p < 0.001, **p < 0.01, *p < 0.05. (**c**) Risk factors for foot pad dermatitis (FPD) in the 2016–2018 dataset. Confidence intervals are 95%. ***p < 0.001, **p < 0.01, *p < 0.05. There were no Hybro breed birds in this analysis. Consistency of performance of farms over time.

Figure 2a–c reports the model results for ascites/oedema, abnormal colour/fevered and foot pad dermatitis in 2016–2018. The model results for other outcomes are presented in Supplementary Figs. S20–S39. The figures depict the standardised regression coefficients along with their 95% confidence intervals for each predictor. If a confidence interval does not overlap with the zero line a predictor can be considered to have a statistically significant impact on the outcome and the p-value is indicated. For Ascites/ Oedema the risk is reduced with Cobb and Hubbard as opposed to Ross birds, more birds in the house, free range and organic production, most months compared to January, stocking density > 39 kg/m^2^, higher maximum temperatures and higher daytime relative humidity (15h00). Increased risks were associated with increasing age at slaughter, increased mortality, stocking density < 33 kg/m^2^, and higher minimum temperatures. abnormal colour/fevered had higher risks associated with increasing age, mortality and flock size, as well as Cobb birds, December and higher minimum temperatures. Most months of the year, stocking density > 39 kg/m^2^ and free-range or organic production types were protective compared to the reference categories, as was higher maximum temperatures and relative humidity at 15h00. By far the largest increase in risk of foot pad dermatitis was for birds in organic production systems, with other smaller increases in free range systems, Cobb birds, increasing age, stocking density > 39 kg/m^2^, November, December, February, March and higher relative humidity at 06h00 and 15h00. Lower FPD risks were associated with Hubbard birds, lower house mortality, stocking density < 33 kg/m^2^, August, September and October.

The proportion of farms in the lower and upper quartiles, and each half of the dataset over time are shown in Table 6. For all measures, a higher proportion of farms were always in Q1 (best performing) than Q4 (worst performing). The proportion in Q4 ranged between 3.00% for joint lesions in 2010–2014 and 11.43% for DOA in 2016–2018.

**Table 6:** Proportion of farms always in each quartile.

Condition	% farms always in quartile			
	Q4	Q3 or Q4	Q1 or Q2	Q1
**2010–2014**				
Ascites/oedema	3.50	11.84	22.44	13.93
Dermatitis cellulitis	4.50	10.09	23.35	16.43
DOA	4.75	7.26	18.77	11.68
Emaciation	4.42	7.76	29.86	15.93
Joint lesions	3.00	6.01	38.28	38.28
Respiratory	4.92	13.01	19.93	11.76
Total rejections	4.67	12.26	13.76	6.26
Mortality house	5.50	13.59	13.84	6.42
**2016–2018**				
Ascites/oedema	9.84	26.36	34.36	19.77
Cellulitis	6.34	24.02	31.69	13.51
Dermatitis	9.26	21.02	25.27	13.51
DOA	11.43	26.02	28.02	15.01
Emaciation	11.09	29.52	31.36	13.84
Joint lesions	8.42	26.86	36.36	36.36
Mortality house	7.42	21.85	26.11	13.09
Mean	6.604	17.16467	26.22667	16.39

### Effect of triggers on adversity reduction

The effect of Type 1 and Type 2 Triggers on subsequent farm performance was tested for time delays of 6, 8, 10, 12 weeks. There was some variability dependent on the length of delay with longer periods of 10 or 12 weeks having larger coefficients, and therefore pointing to a stronger effect than shorter delays. The effect of Triggers on farm performance 10 weeks later is shown in Table 7. In line with the positive interpretation of the change score, higher coefficients indicate a larger effect of that trigger on reducing adverse conditions. All coefficients were significant and show that triggers have a positive effect on reducing adverse conditions. However, it should be recognised that as Trigger farms are already performing poorly there is a greater potential for them to improve their welfare outcome levels than other farms, some of whom may have very low levels of welfare problems (i.e. better welfare) to start with.

**Table 7:** Regression coefficients of effect of Type 1 and Type 2 Triggers on reduction in percentages of conditions after 10 weeks.

Condition	Years	n		Mean % reduction (SE)	
		Batches	Farms	Trigger type 1	Trigger type 2
Ascites/oedema	2010–2014	271,827	1037	0.32 (< 0.001)	1.64 (0.01)
	2016–2018	127,990	1030	3.08 (0.04)	0.34 (0.01)
Dermatitis/cellulitis	2010–2014	271,827	1037	0.06 (< 0.001)	1.90 (0.02)
Cellulitis	2016–2018	127,990	1030	4.67 (0.05)	0.23 (< 0.001)
Dermatitis	2016–2018	127,990	1030	3.84 (0.08)	0.20 (0.01)
Dead on arrival	2010–2014	271,827	1037	0.10 (< 0.001)	1.55 (0.02)
	2016–2018	127,990	1030	2.42 (0.05)	0.10 (0.01)
Emaciation	2010–2014	271,827	1037	0.04 (< 0.001)	0.62 (< 0.001)
	2016–2018	127,990	1030	0.97 (0.01)	0.07 (< 0.001)
Joint lesions	2010–2014	271,827	1037	0.02 (< 0.001)	0.57 (0.01)
	2016–2018	127,990	1030	0.65 (0.01)	0.04 (< 0.001)
Respiratory	2010–2014	271,827	1037	0.50 (0.01)	5.30 (0.06)
Total rejections	2010–2014	271,827	1037	0.95 (0.01)	7.82 (0.09)
Mortality house	2010–2014	271,827	1037	3.28 (0.03)	6.55 (0.08)
	2016–2018	127,990	1030	5.44 (0.11)	N/A
**Mean reduction in FPD score (SE)**					
Foot pad dermatitis	2010–2014	28,105	505	38.16 (2.8)	86.53 (3.52)
	2016–2018	35,701	504	106.62 (3.21)	34.54 (1.46)

All coefficients were significant at the p < 0.001 level.

Table 8 shows that for all outcomes in at least one time period, with the exception of Mortality House, the proportion of triggers issued in the previous 3 months is significantly related to the odds of moving out of the 4th quartile. However, the odds ratios, which are all below one, show that this relationship is negative and therefore a reduced likelihood of moving to the 3rd or lower quartile for farms with a larger proportion of triggers received in the previous time window.

**Table 8:** The odds of a poorly performing farm moving from the fourth quartile in the preceding time window into a different quartile 3 months later as a function of the proportion of triggers issued in the preceding time window.

Condition			Odds ratio	Log (OR)	SE Log (OR)	p
Ascites/oedema	2010–2014	Intercept	2.26	0.81	0.21	< 0.001
		Trigger 1	0.38	− 0.97	0.25	< 0.001
	2016–2018	Intercept	1.38	0.32	25.00	0.20
		Trigger 1	0.27	− 1.29	0.17	< 0.001
Dermatitis cellulitis	2010–2014	Intercept	3.28	1.19	0.15	< 0.001
		Trigger 1	0.15	− 1.92	0.18	< 0.001
Cellulitis	2016–2018	Intercept	0.63	− 0.46	0.14	< 0.001
		Trigger 1	0.37	− 0.98	0.13	< 0.001
Dermatitis	2016–2018	Intercept	0.41	− 0.89	0.07	< 0.001
		Trigger 1	0.33	− 1.10	0.28	< 0.001
DOA	2010–2014	Intercept	5.75	1.75	0.21	< 0.001
		Trigger 1	0.15	− 1.89	0.26	< 0.001
	2016–2018	Intercept	1.93	0.66	0.13	< 0.001
		Trigger 1	0.15	− 1.90	0.13	< 0.001
Emaciation	2010–2014	Intercept	4.68	1.54	0.19	< 0.001
		Trigger 1	0.09	− 2.46	0.24	< 0.001
	2016–2018	Intercept	0.93	− 0.07	0.11	0.53
		Trigger 1	0.11	− 2.18	0.16	< 0.001
Joint lesions	2010–2014	Intercept	1.64	0.49	0.15	< 0.001
		Trigger 1	0.33	− 1.12	0.23	< 0.001
	2016–2018	Intercept	0.79	− 0.24	0.18	0.20
		Trigger 1	0.29	− 1.25	0.05	< 0.001
Respiratory	2010–2014	Intercept	2.60	0.96	0.13	< 0.001
		Trigger 1	0.22	− 1.54	0.21	< 0.001
Total rejections	2010–2014	Intercept	4.17	1.43	0.20	< 0.001
		Trigger 1	0.23	− 1.46	0.25	< 0.001
Mortality house	2010–2014	Intercept	1.22	0.20	0.07	< 0.001
		Trigger 1	0.66	− 0.42	0.25	0.09
	2016–2018	Intercept	0.41	− 0.89	0.06	< 0.001
		Trigger 1	0.69	− 0.37	1.10	0.73

## Discussion

This huge dataset, representing 3.1 billion broilers, provides an opportunity for comprehensive analysis rarely available outside national monitoring organisations, including those which are privately administered, such as a farm assurance schemes^11^, or co-ordinated by public bodies^12^. The analysis provides new insights into the performance of individual UK broiler farms over a prolonged period of time, as well as a greater understanding of risk factors for measures of poor health and welfare detected through systematic and centrally reported slaughterhouse observations.

There were some practical limitations of this study due to the retrospective nature of the data provided, limited ability to interrogate procedures surrounding data collection, and the issue of missing data and subsequent changes in categorising outcomes. In addition, the exploratory nature of the analysis resulted in a large number of models which is likely to have led to some false positive results, although we minimised the chance of this occurring through pursuing only the most robust models, using magnitude of coefficients and their confidence intervals rather than p-values to drive our inferences and not performing post-hoc analysis. However, the capacity to identify farms most likely to have poor welfare is an important strategy for improving animal welfare overall, and for maximising the capacity for checking regulatory compliance when resources are limited. That a greater proportion of broiler farms overall remained consistently in the best quartile (17%) rather than the worst quartile (7%) is heartening. Currently in the UK these ‘consistently better’ farms are not rewarded by, for example, reduced frequency or scope of farm assurance visits, although the use of risk-based assessment intervals is being reviewed by some farm assurance schemes. Some companies and retailers have their own requirements for higher resource provision and it is not known from this data source whether these ‘consistently better’ farms are part of a these ‘higher welfare’ systems, schemes which may in turn provide rewards in other ways, such as improved price or security to supply.

Regulatory monitoring of broiler welfare in the UK to comply with the European Broiler Directive^1^ is currently targeted mainly at farms that exceed a ‘trigger’ threshold, and so indicate risk of poor welfare as indicated by measures monitored by slaughterhouses (see Table 2). These trigger farms are already performing ‘very poorly’ when compared to their peers. We found that these ‘trigger farms’ were more likely to improve after exceeding a trigger threshold than other farms, however, it should be noted that this is a likely outcome (reversion to the mean, i.e. improvement) when the initial starting point is very low. It is clear from the data analysis however, that although these farms do, on the whole, improve, the low performance and hence the raising of triggers, are not, in general, the result of an aberration of an otherwise good farm. Rather, these farms creep out of the trigger zone but tend to remain in the worst performing quartile for all farms. From these results it is reasonable to continue to target trigger farms to improve, as well as to continue to monitor farms consistently in the worst quartile, even if they do not breach trigger thresholds. The mechanisms available to provide support include via veterinary surgeons, companies and through public or private industry bodies, all of which can be encouraged by government initiatives.

The practical enforcement implication of breaching the trigger threshold changed during the course of time for the data that we analysed. Initially (2010–2014) a paper report was followed up by phone calls and visits from APHA staff. In the latter period (2016–2018) the use of a paper report to the producer continued, but there was a reduction in the frequency of additional phone-calls, and an increase in use of targeted visits by official inspectors^13^. We were unable to analyse the impact of these practical enforcement and support changes, but we stress that further understanding of the most effective, as well as cost-effective, methods to bring about welfare improvements is important. A study of the impact of regulatory controls on poorly performing dairy farms in France found that two visits were required to demonstrate improved welfare^14^, although there was only a 23% chance that these farms would improve. Both Lomellini-Dereclenne et al.^14^ and Kelly et al.^15^ identified a number of other farmer activities as risk factors for regulatory non-compliance, and improving understanding of farmer intent and possible actions could be a focus of further research activities on UK broiler farms.

Only Foot Pad Dermatitis (FPD) appeared to improve substantially over the time period analysed, where there was a step change in reported values following the data interruption, from a mean score of 71.9 for the period 2010–2014 and 25.1 for 2016–2018 out of a possible maximum of 200. The limited other reported data sources for this time period appear to corroborate the step change in values as a genuine improvement rather than, for example, an altered recording methodology. The UK retailer Waitrose, utilising higher welfare systems, reports a mean proportion of birds affected by FPD reducing from 57% for 2011–2014 to 14% for 2016–2018^16^. In addition, the food business KFC reports for its UK and Ireland supply chain an improvement from 57% of birds affected by FPD in 2015 to 36% affected in 2018^17^. It seems at least some sections of the broiler industry attempted to tackle high levels of FPD around 2014 with the introduction of financial incentives to farmers for low levels of FPD, and promotion of a switch to biomass boilers resulting in better litter quality (personal communication C. Willson, Food Standards Agency).

The risk factors for individual welfare measures that were identified in this study may allow targeted mitigating action to be taken. Some measures were found to be highly seasonal and to be profoundly affected by the weather, including ascites, the most frequently occurring single condition (0.38% in 2016–2018). Ascites was found to be significantly worse during colder weather, and exposure to colder temperatures in the house, or poor ventilation associated with preservation of in house temperature by reduction in fan ventilation rates, has previously been found to be a risk factor^18^ suggesting better temperature and ventilation control in the houses may help to reduce ascites levels. Part et al.^6^ suggested that climate change may have substantial impacts on broiler welfare and productivity and that modelling such effects should be a focus of future research in order to promote housing and management practices to counter the negative consequences of such changes.

In comparison to Ross birds, Cobb birds had higher risk of abnormal colour/ fever and FPD but a lower risk of ascites/ oedema and Hubbard birds had lower risk of all three conditions. Few direct breed comparisons are published, and sometimes for commercially sensitive reasons the breeds are not named (e.g. Rayner et al.^19^). For ascites, a condition resulting from cardiac/ circulatory insufficiency^20^, a small-scale pen trial compared, amongst others, Cobb, Ross and Hubbard breeds and found no significant difference in heart traits and mortality at 42 days between breeds. It is likely that integrator and breeding companies have data on the performance of breeds that is not published. That free range and organic systems showed lower risk than extensive indoor systems for both ascites and abnormal colour is interesting but difficult to explain given that breed and stocking density were already accounted for in the model. Extensive indoor systems did not show better health outcomes in our study, likely partly influenced by the relatively low numbers of such farms in our sample. Extensive indoor systems tend to use slower growing breeds and offer more space for the birds, both of which have been found in other studies to have better health outcomes than the breeds and stocking densities commonly found in intensive indoor systems^19,21^. The increased risk of FPD in free range or organic systems is consistent with previous findings, however some of this difference has been suggested to be accounted for by inapplicability and inconsistency of scoring systems for organic birds^22^ and higher levels of biologically less significant hyperkeratosis in organic compared to conventional birds^23^.

This study confirms that continuous monitoring of large scale slaughterhouse derived data can provide a useful tool for regulatory activities as well as helping to drive industry changes which result in improved welfare conditions for farmed broiler chicken.

## Supplementary Information

Supplementary Information.
