# Supplementary material for: Longitudinal national-level monitoring of on-farm broiler welfare identifies consistently poorly performing farms
Source: Sci Rep. 2021 Jun 7;11:11928. doi: 10.1038/s41598-021-91347-4 (PMC8185078; doi:10.1038/s41598-021-91347-4)
Supplement: Supplementary file 1 — Supplementary Information. [file 41598_2021_91347_MOESM1_ESM.docx]

Longitudinal national-level monitoring of on-farm broiler welfare identifies consistently poorly performing farmsAuthors: Siobhan Mullan*, Bobby Stuijfzand and Andrew Butterworth

Bristol Veterinary School, University of Bristol, Langford, Avon, UK, BS40 5DU

*Corresponding Author: Siobhan.mullan@ucd.ie

Supplementary material

Figures S1 to S16: The daily mean outcomes over time. Note some measures were only recorded in one time period or another (2010-2014 or 2016-2018) and that there is no data for any measure between 9^th^ June 2014 and 1^st^ April 2016.

%


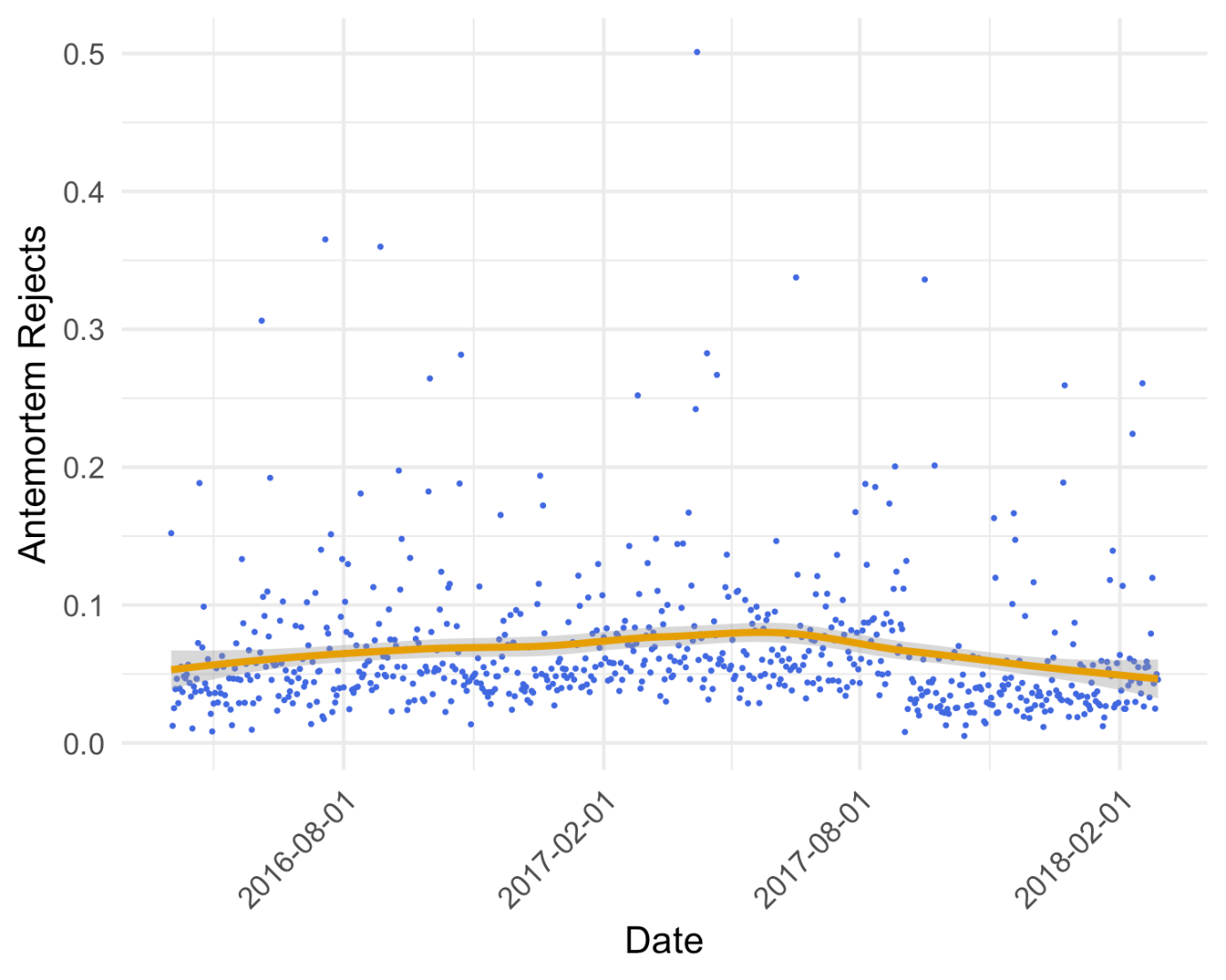


Figure S1. Daily Mean Percentage of Birds Rejected Antemortem, represented by each dot. The line is the smoothing function to visualise trends (Local Polynomial Regression fitting also known as LOESS) and associated shading denotes the 95% confidence interval


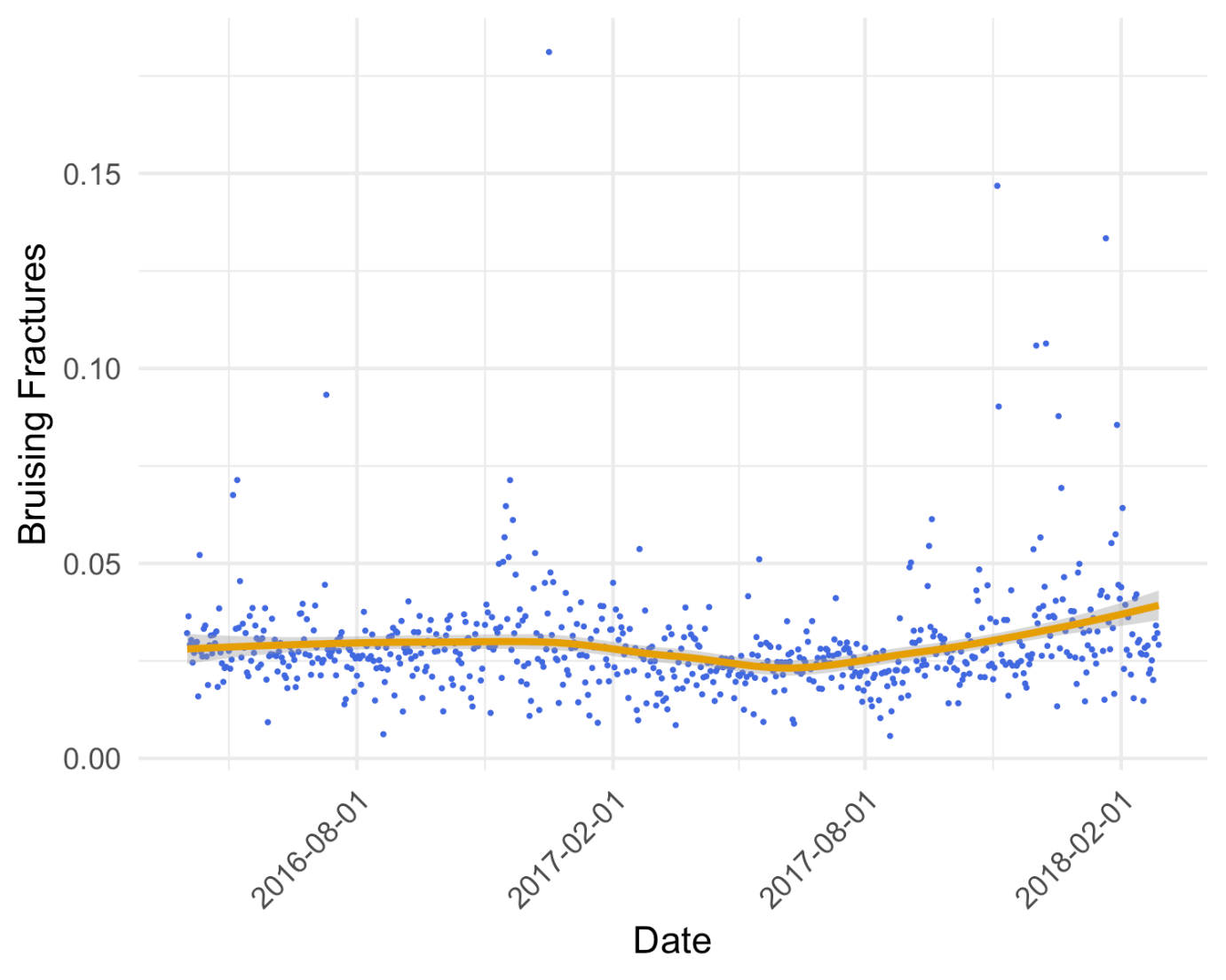


%

Figure S2. Daily Mean Percentage of Birds with Bruisings and Fractures, represented by each dot. The line is the smoothing function to visualise trends (Local Polynomial Regression fitting also known as LOESS) and associated shading denotes the 95% confidence interval


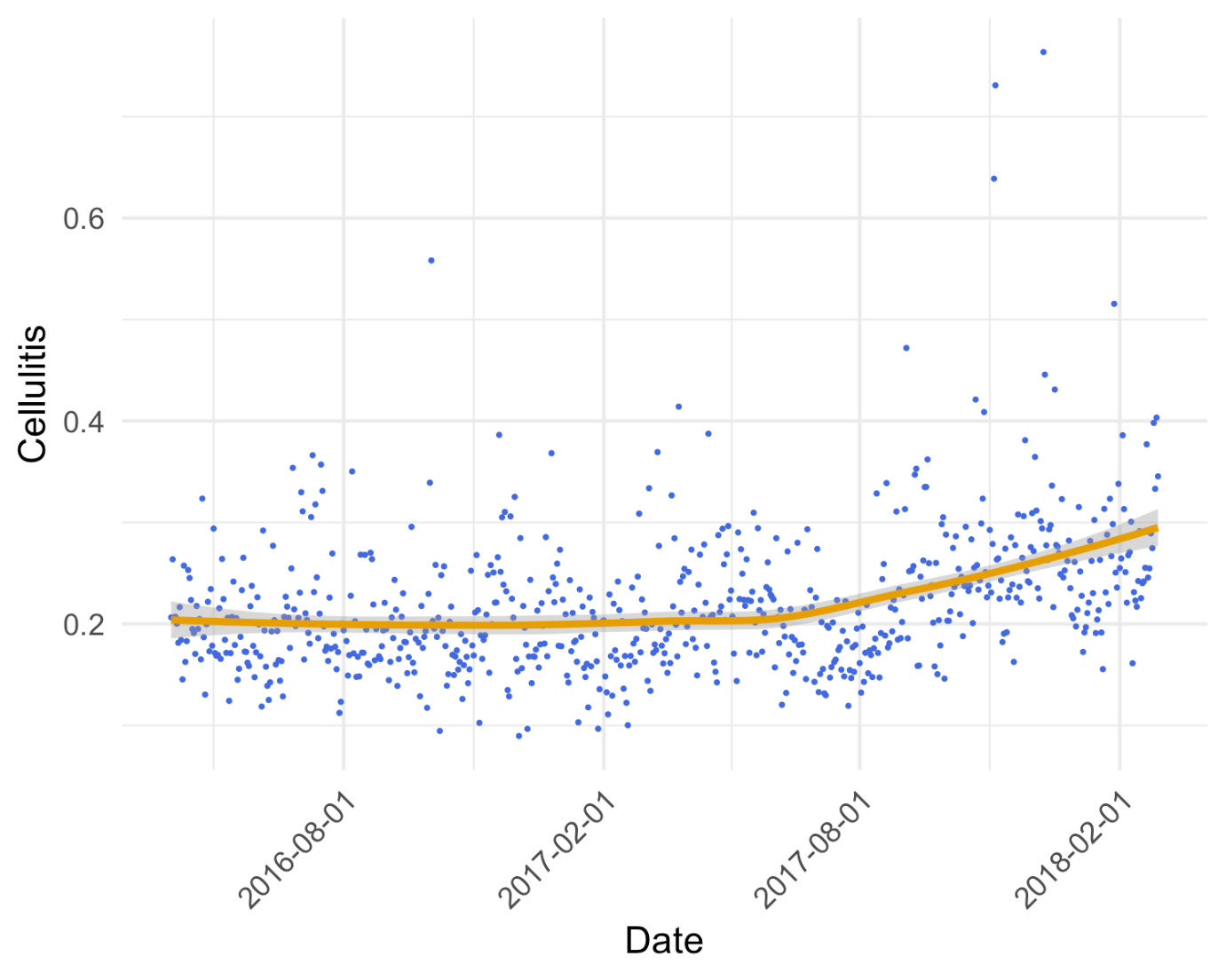


%

Figure S3. Daily Mean Percentage of Birds with Cellulitis, represented by each dot. The line is the smoothing function to visualise trends (Local Polynomial Regression fitting also known as LOESS) and associated shading denotes the 95% confidence interval


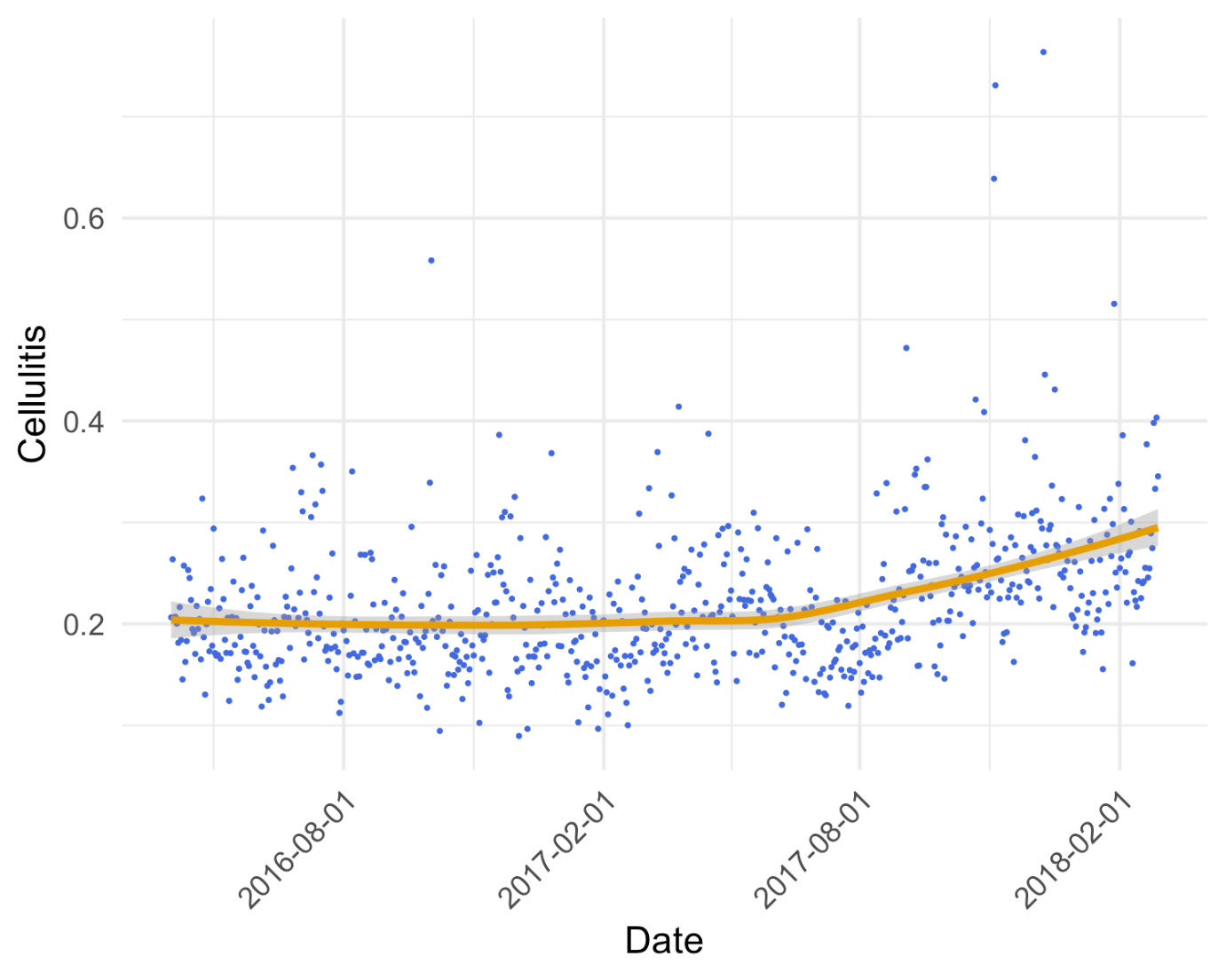


%

Figure S4. Daily Mean Percentage of Birds with Dermatitis and Cellulitis, represented by each dot. The line is the smoothing function to visualise trends (Local Polynomial Regression fitting also known as LOESS) and associated shading denotes the 95% confidence interval


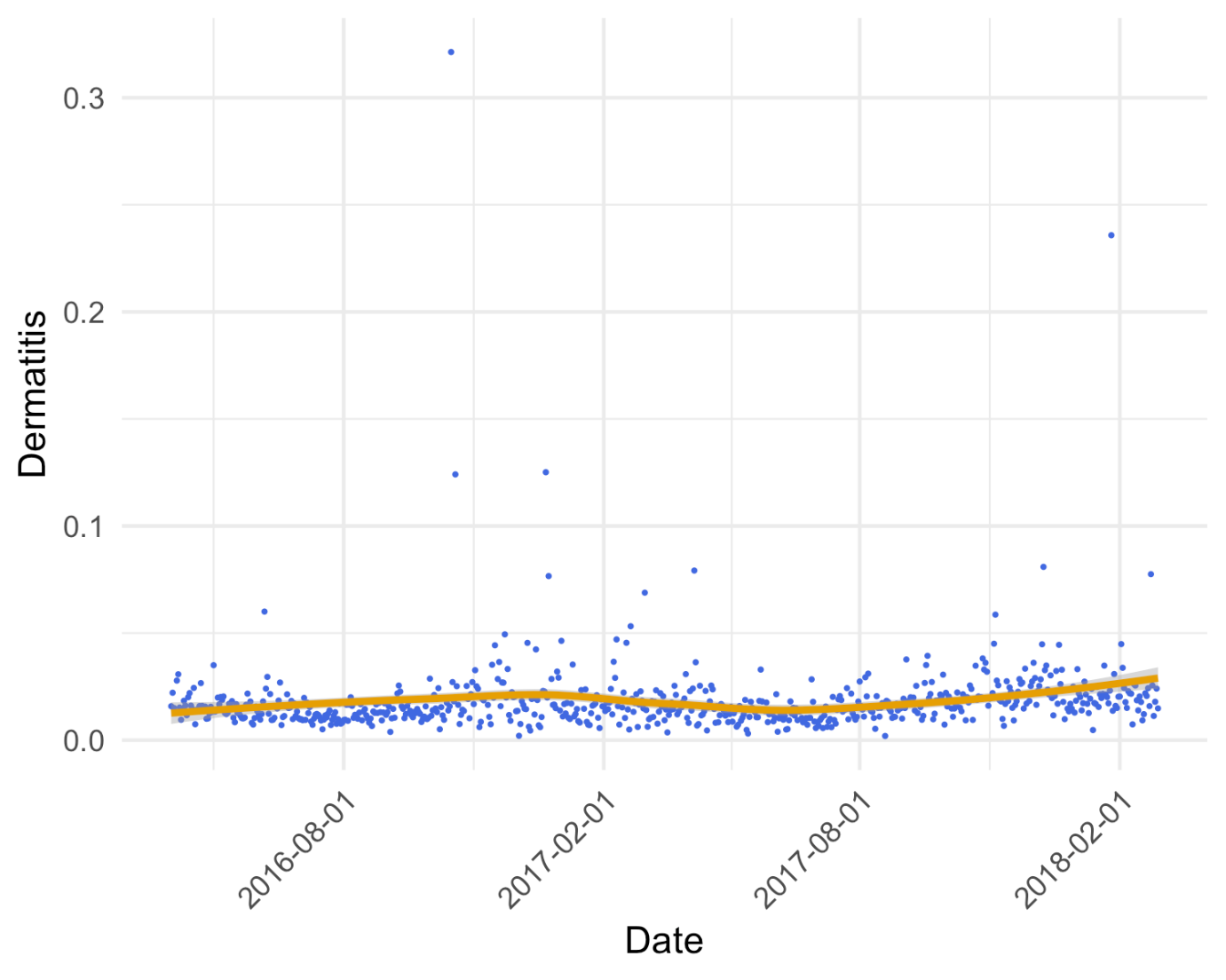


%

Figure S5. Daily Mean Percentage of Birds with Dermatitis, represented by each dot. The line is the smoothing function to visualise trends (Local Polynomial Regression fitting also known as LOESS) and associated shading denotes the 95% confidence interval


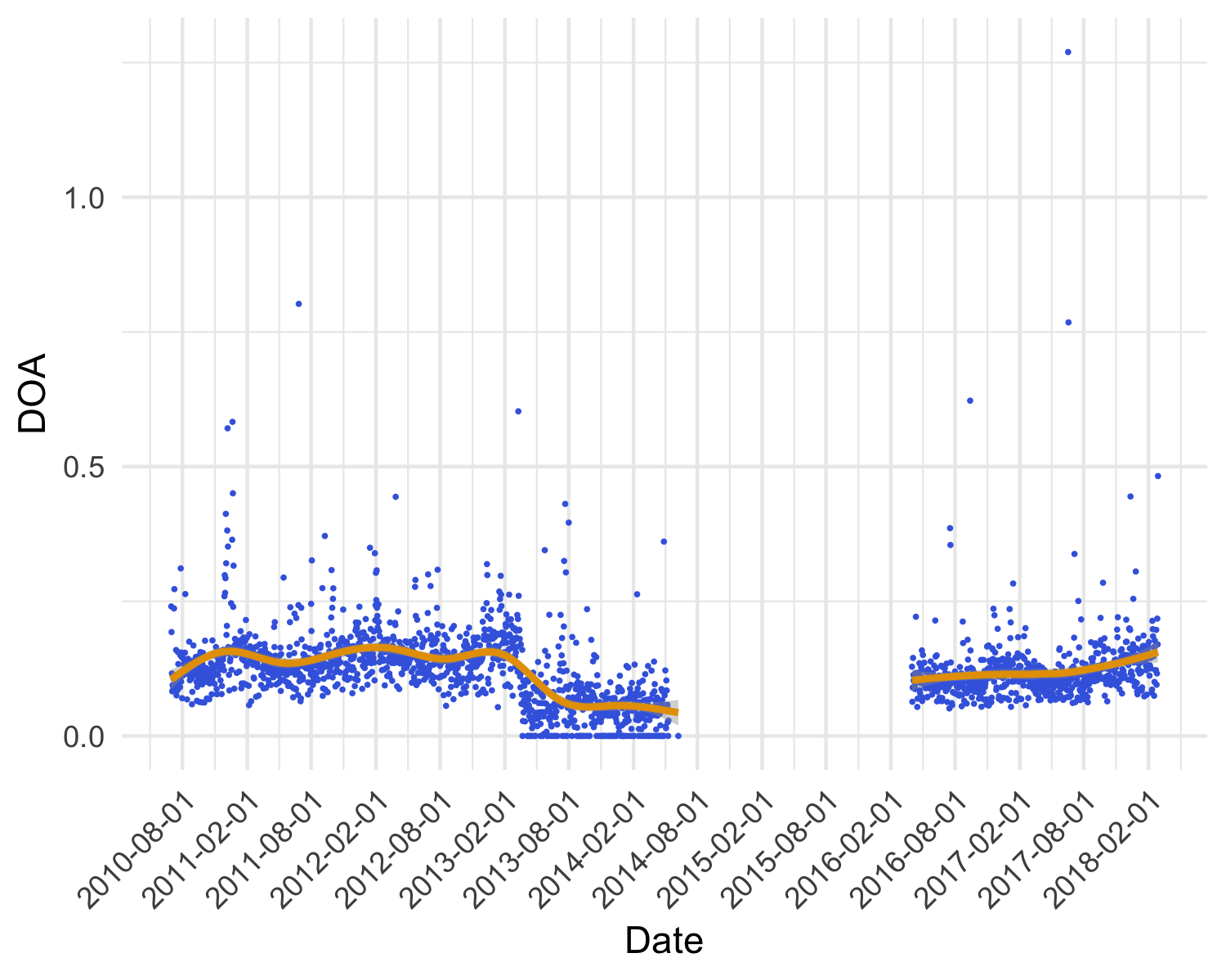


%

Figure S6. Daily Mean Percentage of Birds Dead on Arrival, represented by each dot. The line is the smoothing function to visualise trends (Local Polynomial Regression fitting also known as LOESS) and associated shading denotes the 95% confidence interval


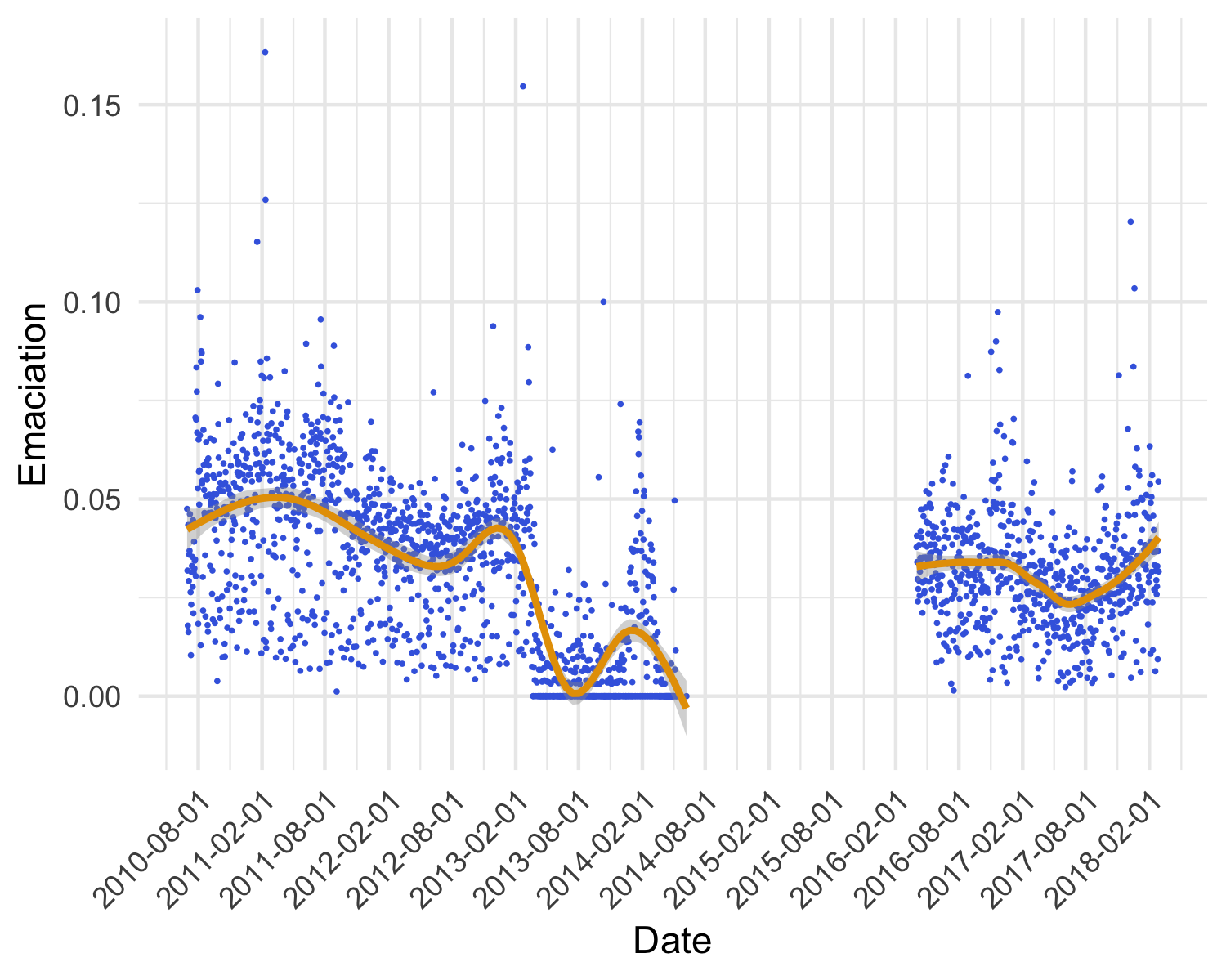


%

Figure S7. Daily Mean Percentage of Birds with Emaciation, represented by each dot. The line is the smoothing function to visualise trends (Local Polynomial Regression fitting also known as LOESS) and associated shading denotes the 95% confidence interval


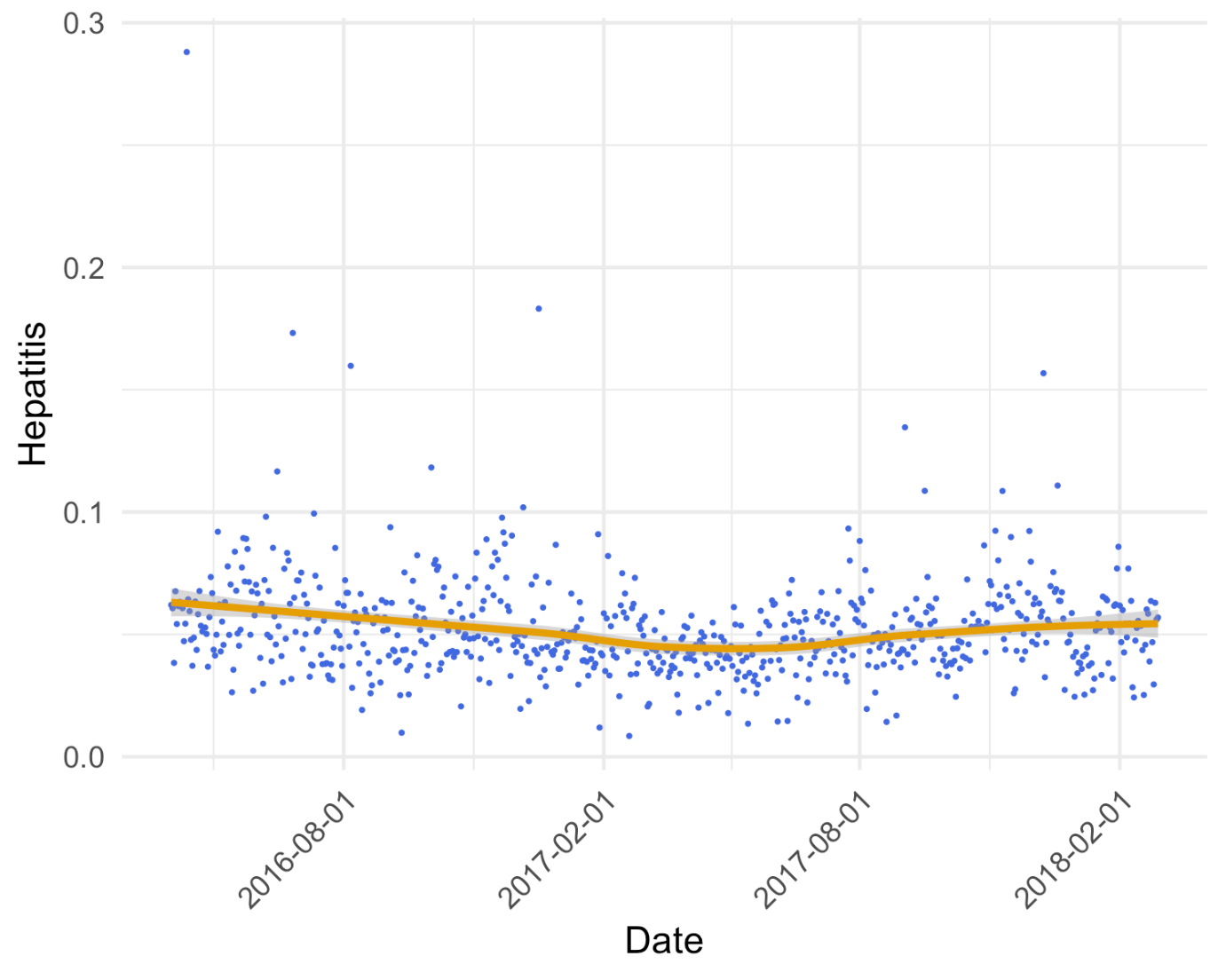


%

Figure S8. Daily Mean Percentage of Birds with Hepatitis, represented by each dot. The line is the smoothing function to visualise trends (Local Polynomial Regression fitting also known as LOESS) and associated shading denotes the 95% confidence interval


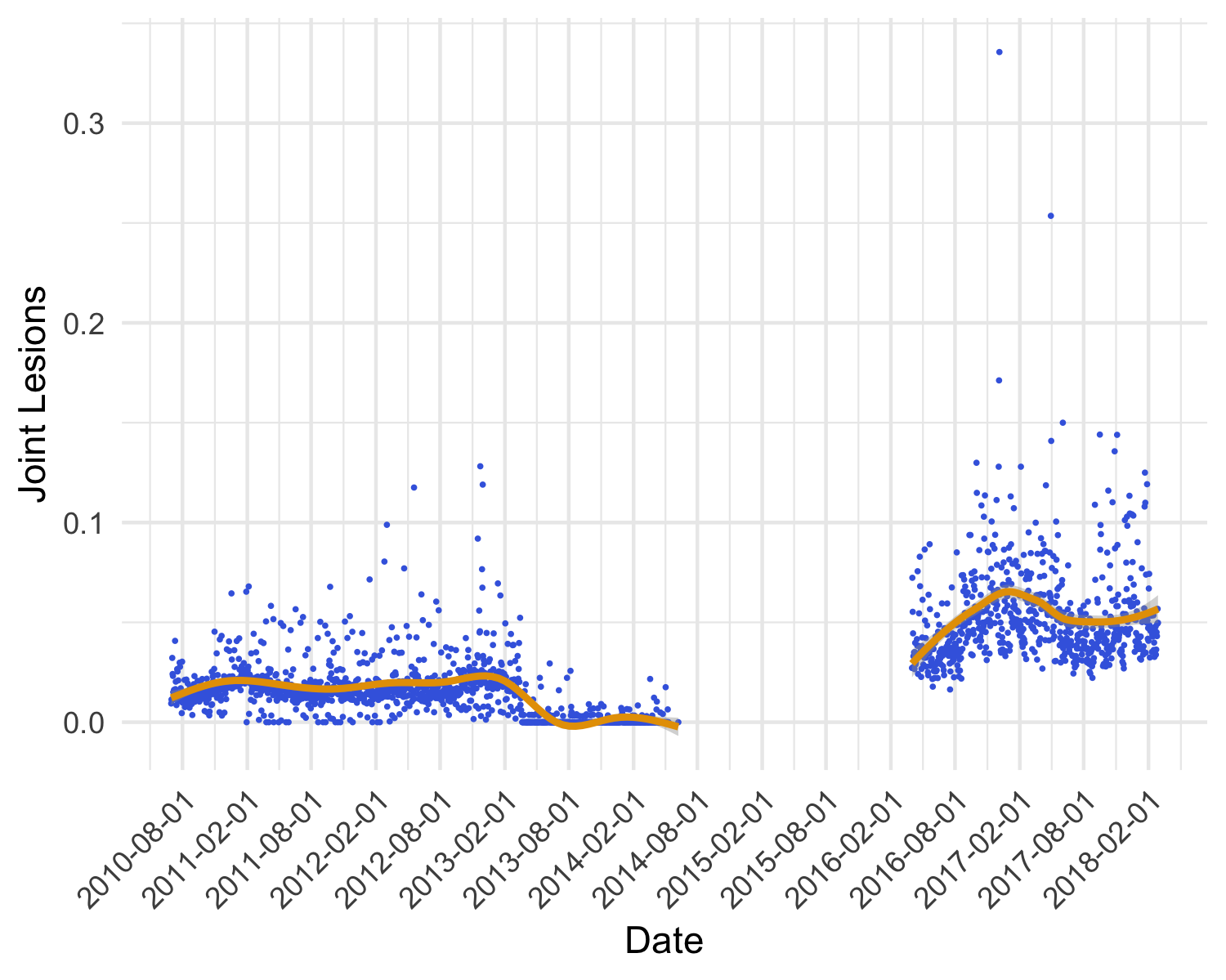


%

Figure S9. Daily Mean Percentage of Birds with Joint Lesions, represented by each dot. The line is the smoothing function to visualise trends (Local Polynomial Regression fitting also known as LOESS) and associated shading denotes the 95% confidence interval


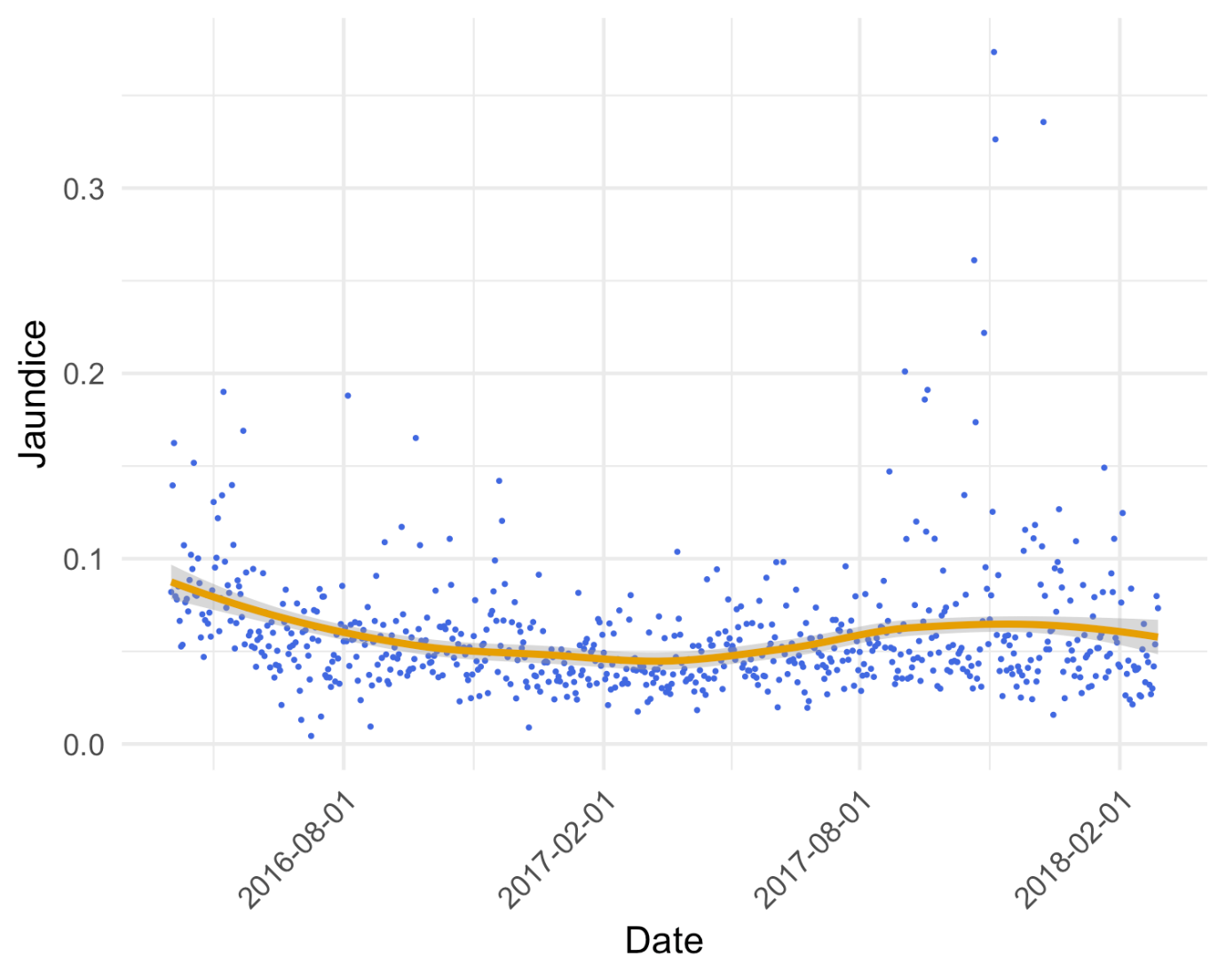


%

Figure S10. Daily Mean Percentage of Birds with Other Farm Jaundice, represented by each dot. The line is the smoothing function to visualise trends (Local Polynomial Regression fitting also known as LOESS) and associated shading denotes the 95% confidence interval


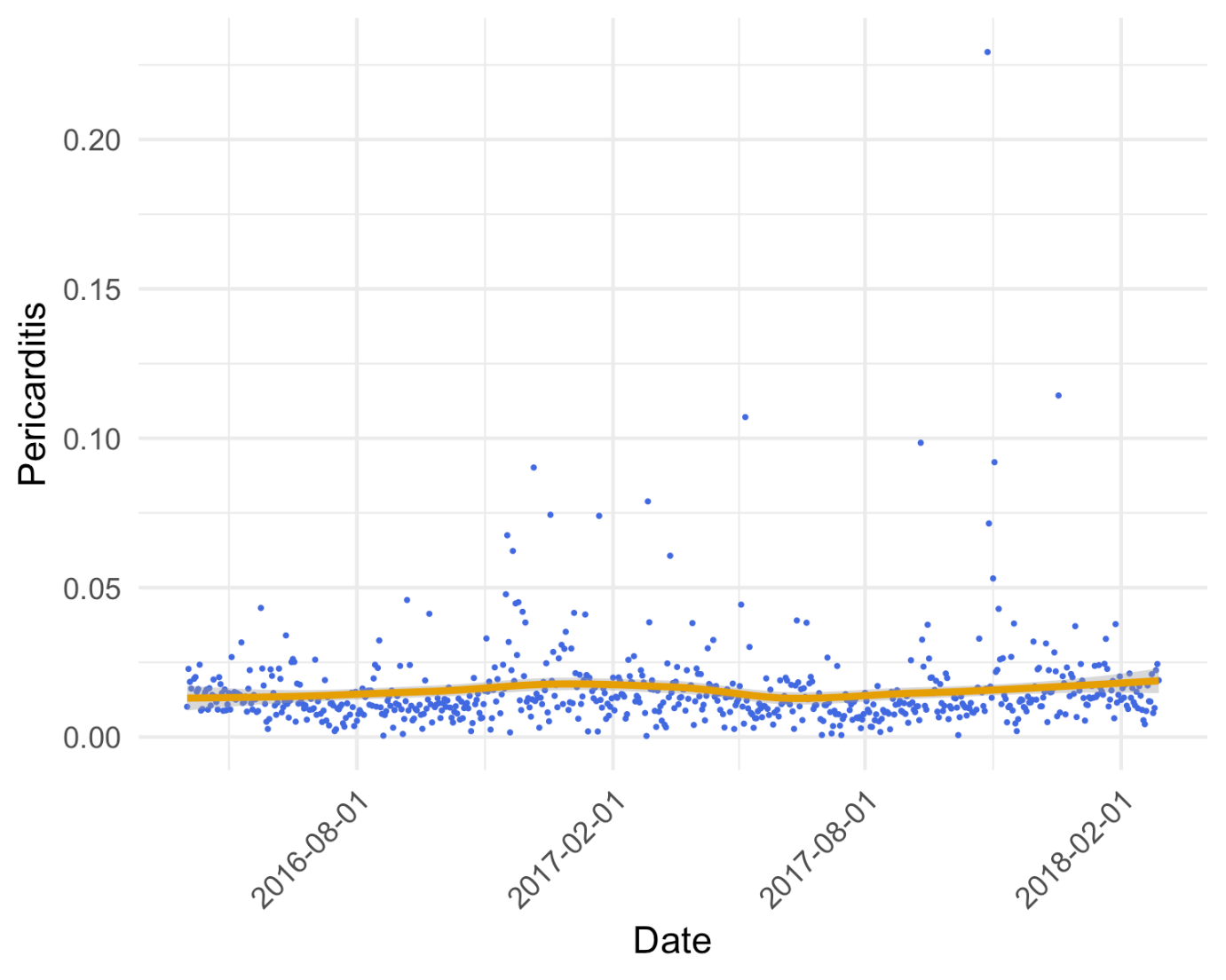


%

Figure S11. Daily Mean Percentage of Birds with Pericarditis, represented by each dot. The line is the smoothing function to visualise trends (Local Polynomial Regression fitting also known as LOESS) and associated shading denotes the 95% confidence interval


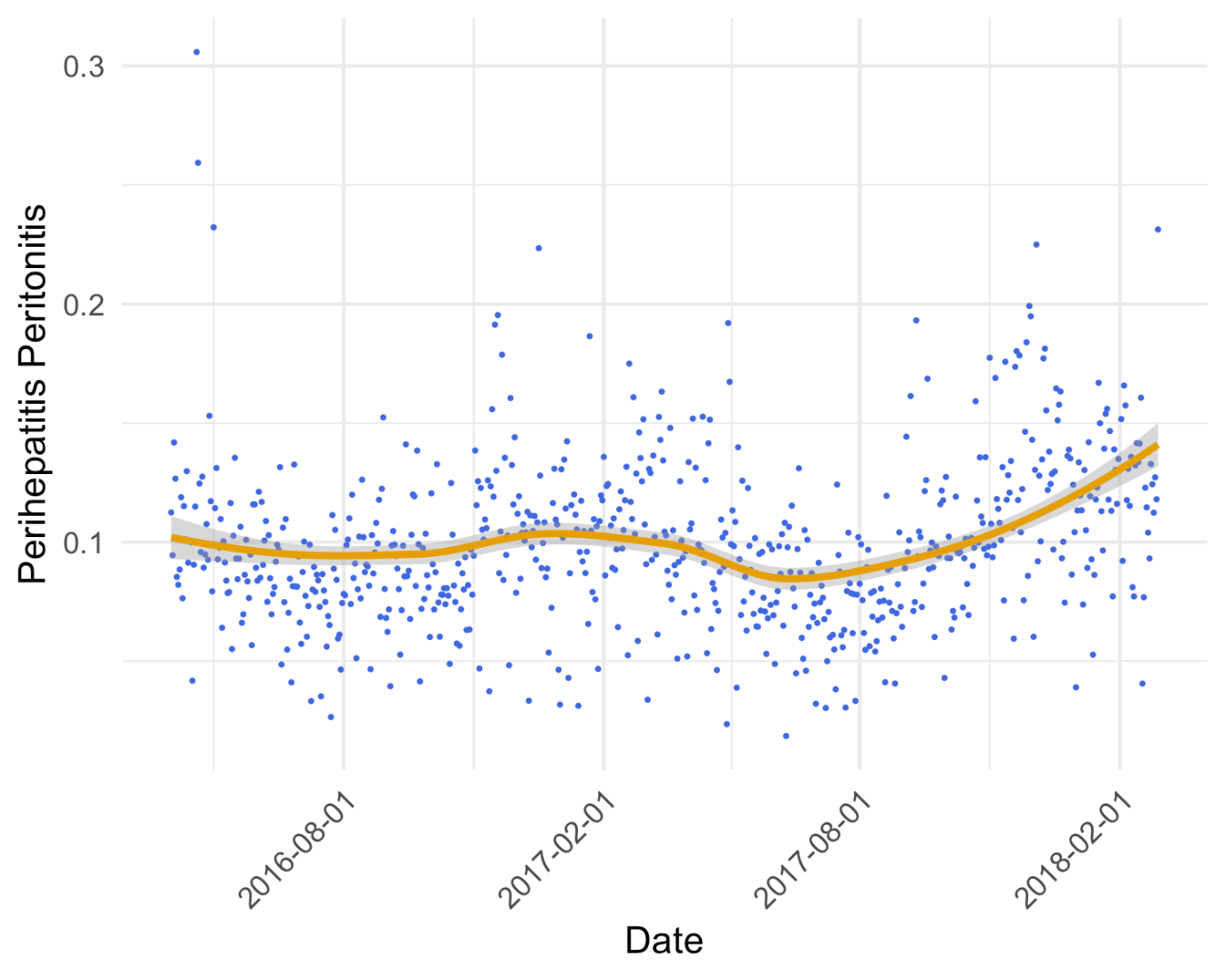


%

Figure S12. Daily Mean Percentage of Birds with Perihepatitis Peritonitis, represented by each dot. The line is the smoothing function to visualise trends (Local Polynomial Regression fitting also known as LOESS) and associated shading denotes the 95% confidence interval


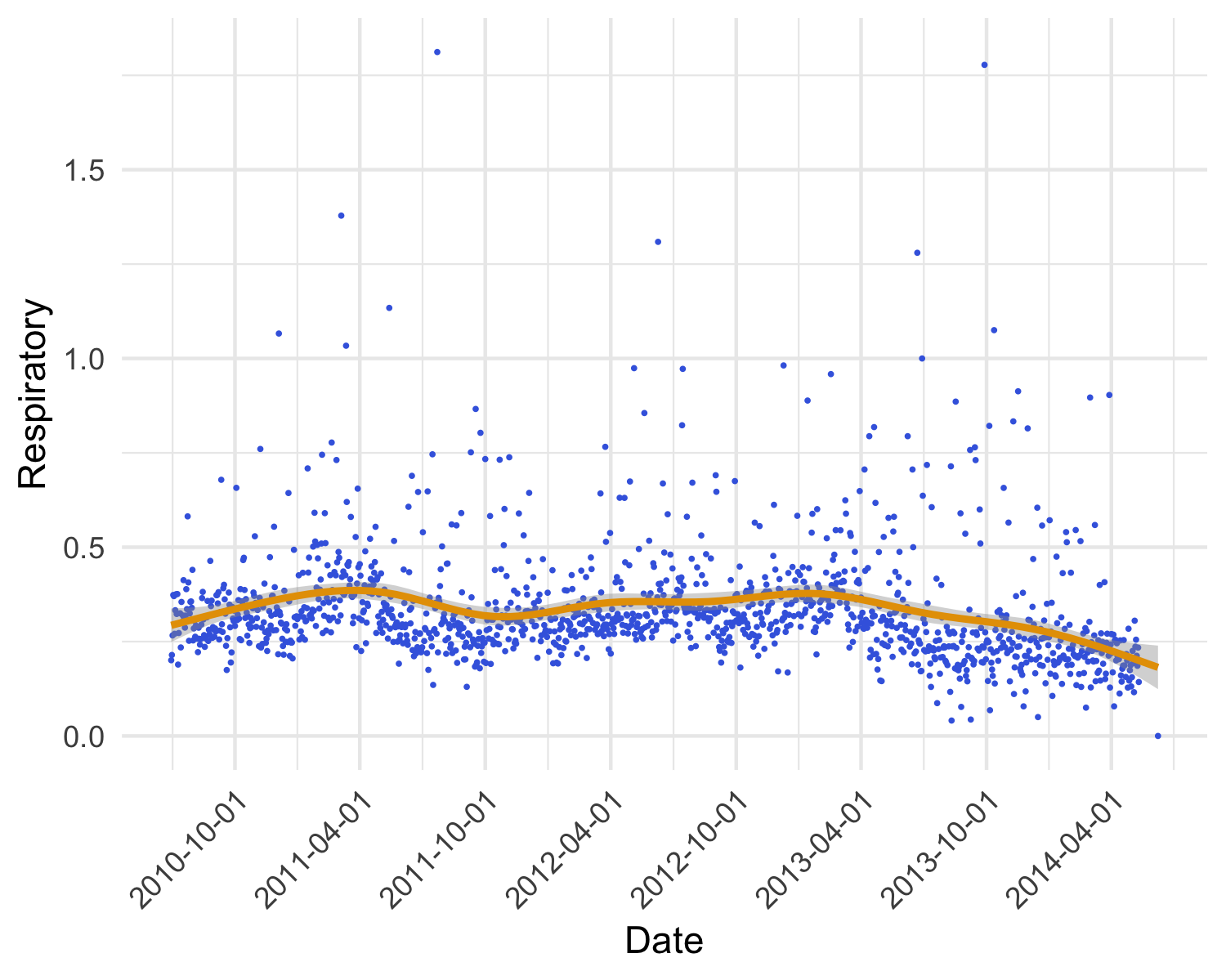


%

Figure S13. Daily Mean Percentage of Birds with Respiratory Disease, represented by each dot. The line is the smoothing function to visualise trends (Local Polynomial Regression fitting also known as LOESS) and associated shading denotes the 95% confidence interval


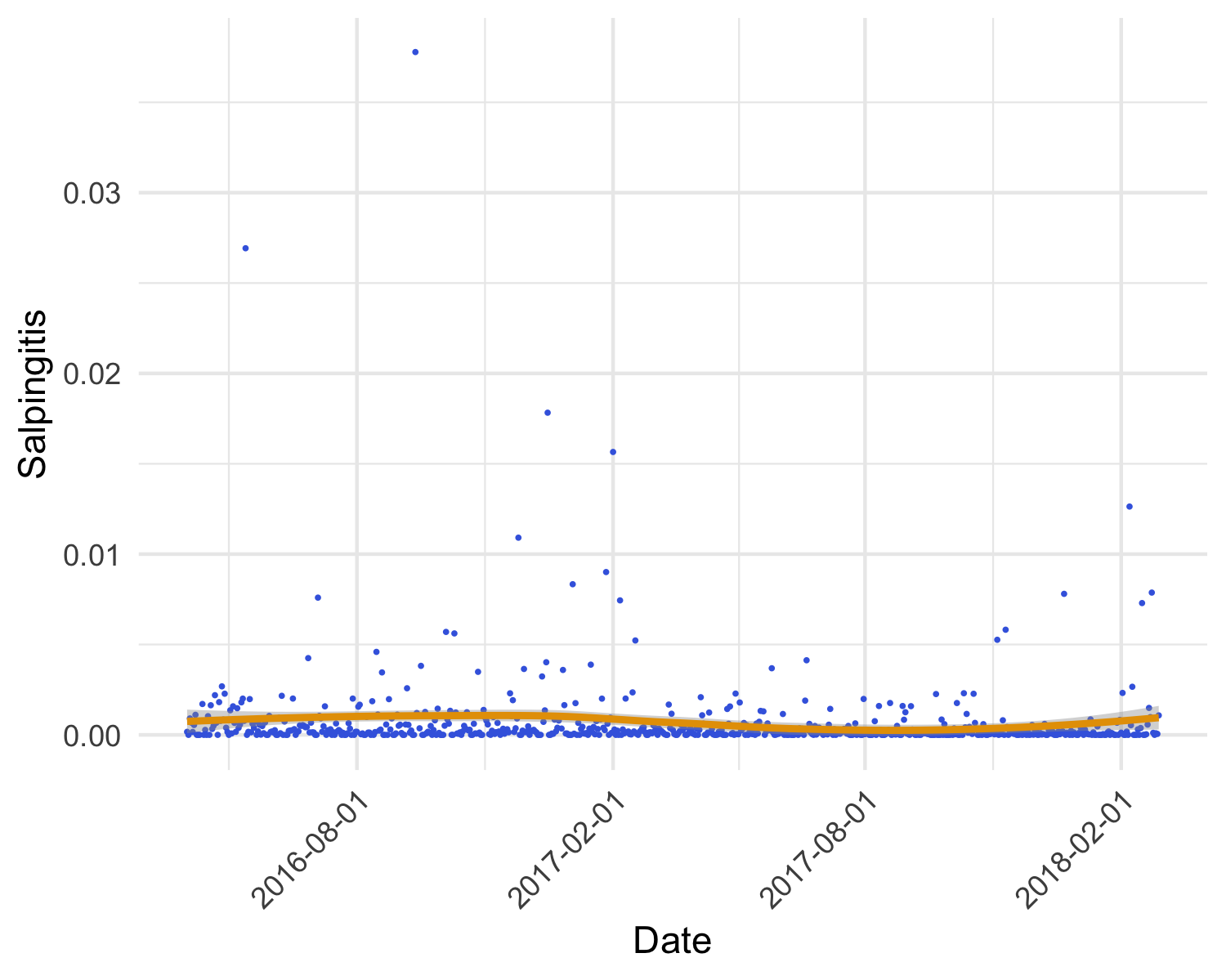


%

Figure S14 Daily Mean Percentage of Birds with Salpingitis, represented by each dot. The line is the smoothing function to visualise trends (Local Polynomial Regression fitting also known as LOESS) and associated shading denotes the 95% confidence interval


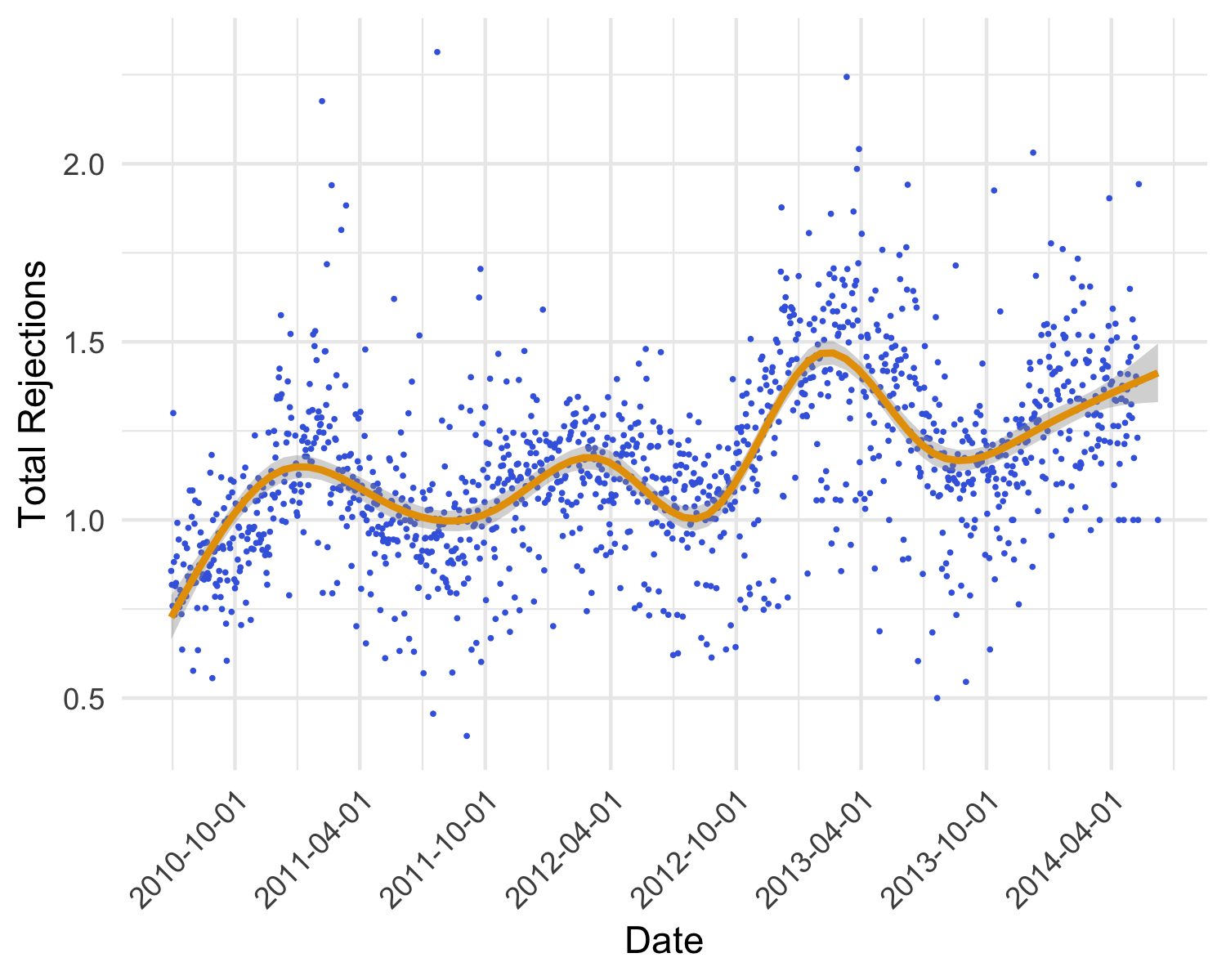


%

Figure S15. Daily Mean Percentage of Birds Rejected, represented by each dot. The line is the smoothing function to visualise trends (Local Polynomial Regression fitting also known as LOESS) and associated shading denotes the 95% confidence interval


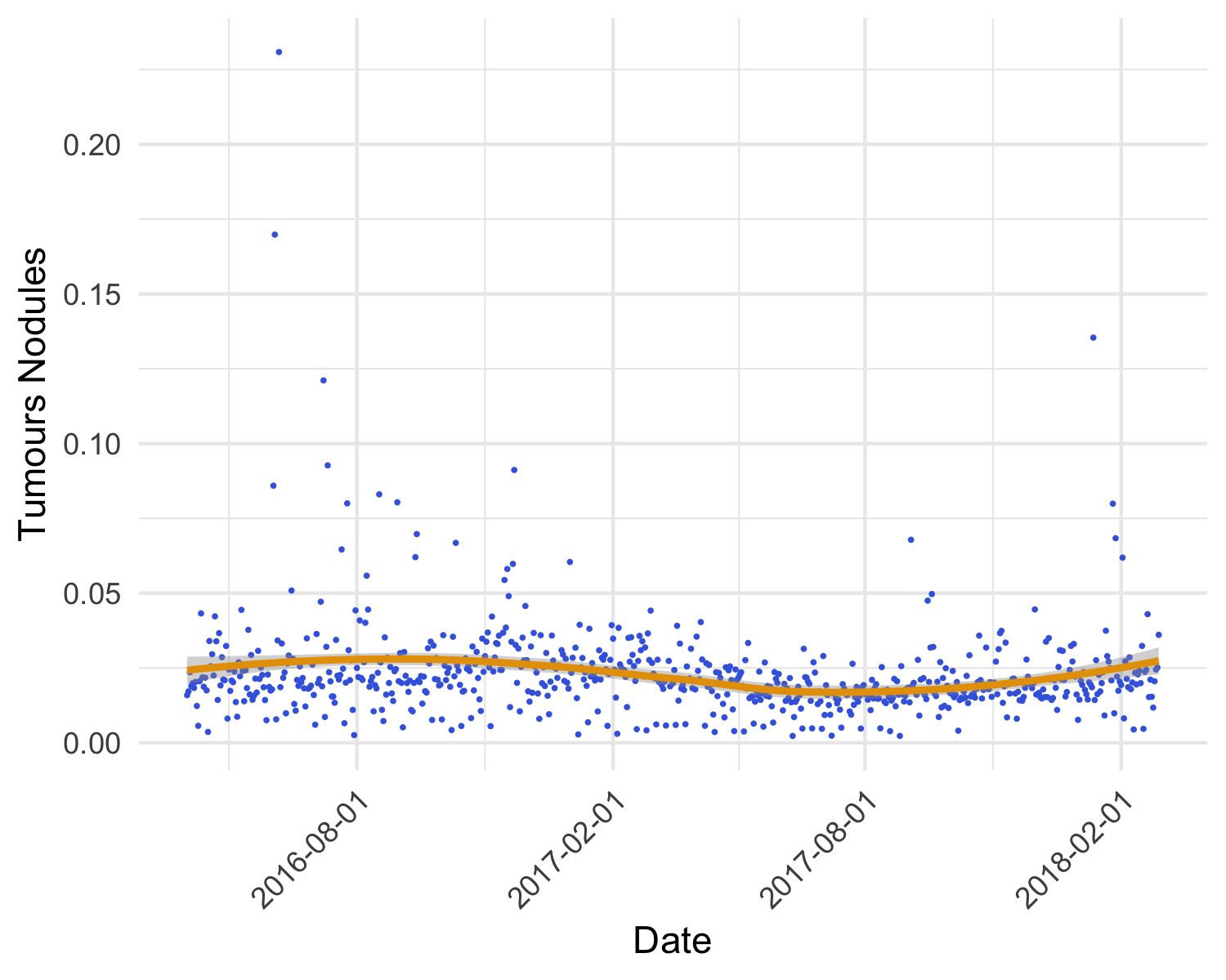


%

Figure S16. Daily Mean Percentage of Birds with Tumours/ Nodules, represented by each dot. The line is the smoothing function to visualise trends (Local Polynomial Regression fitting also known as LOESS) and associated shading denotes the 95% confidence interval

Figures S17-19. Explained variance for factors included in the model


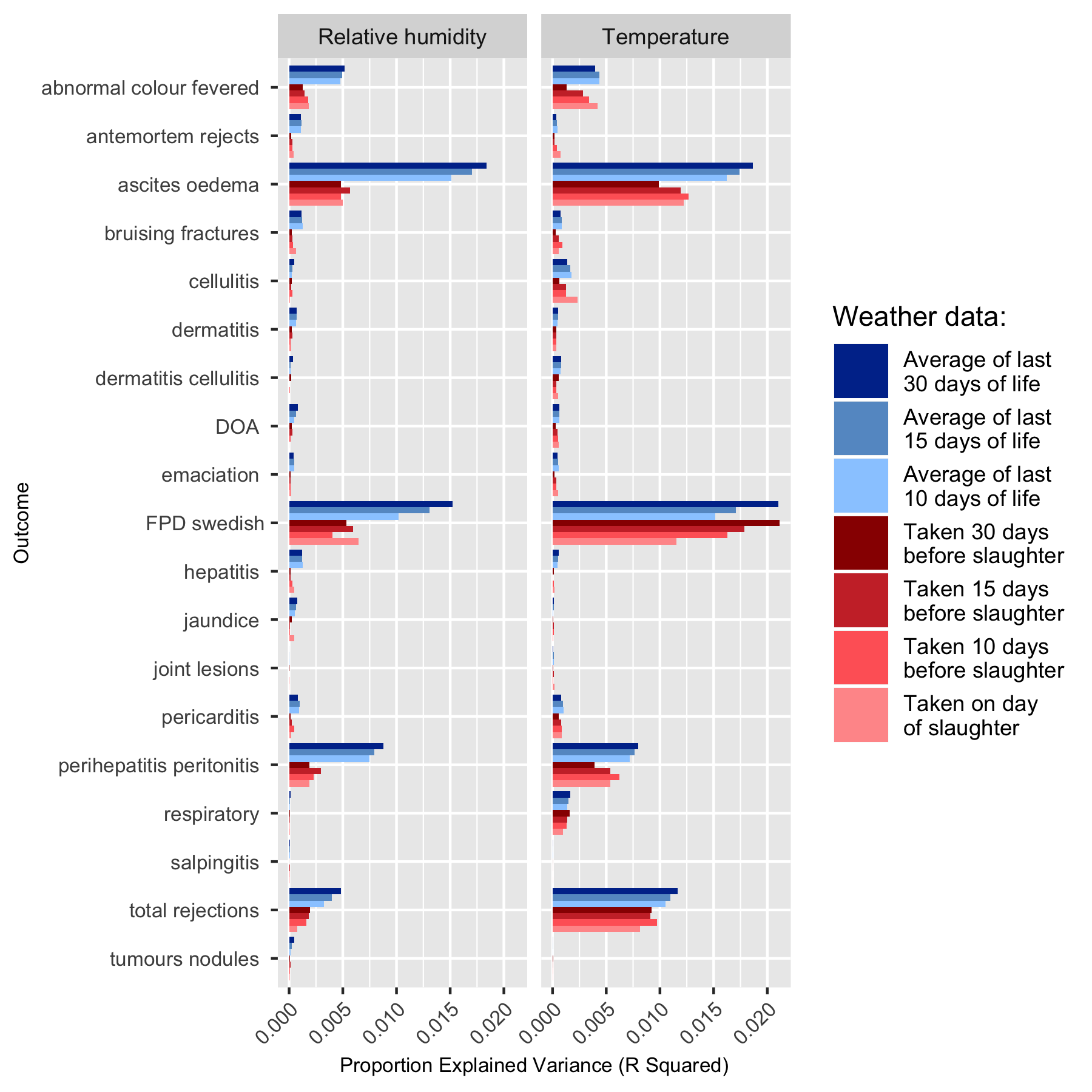


Figure S17. Explained Variance in Each Outcome for Weather Variables Covering Different Periods of Time


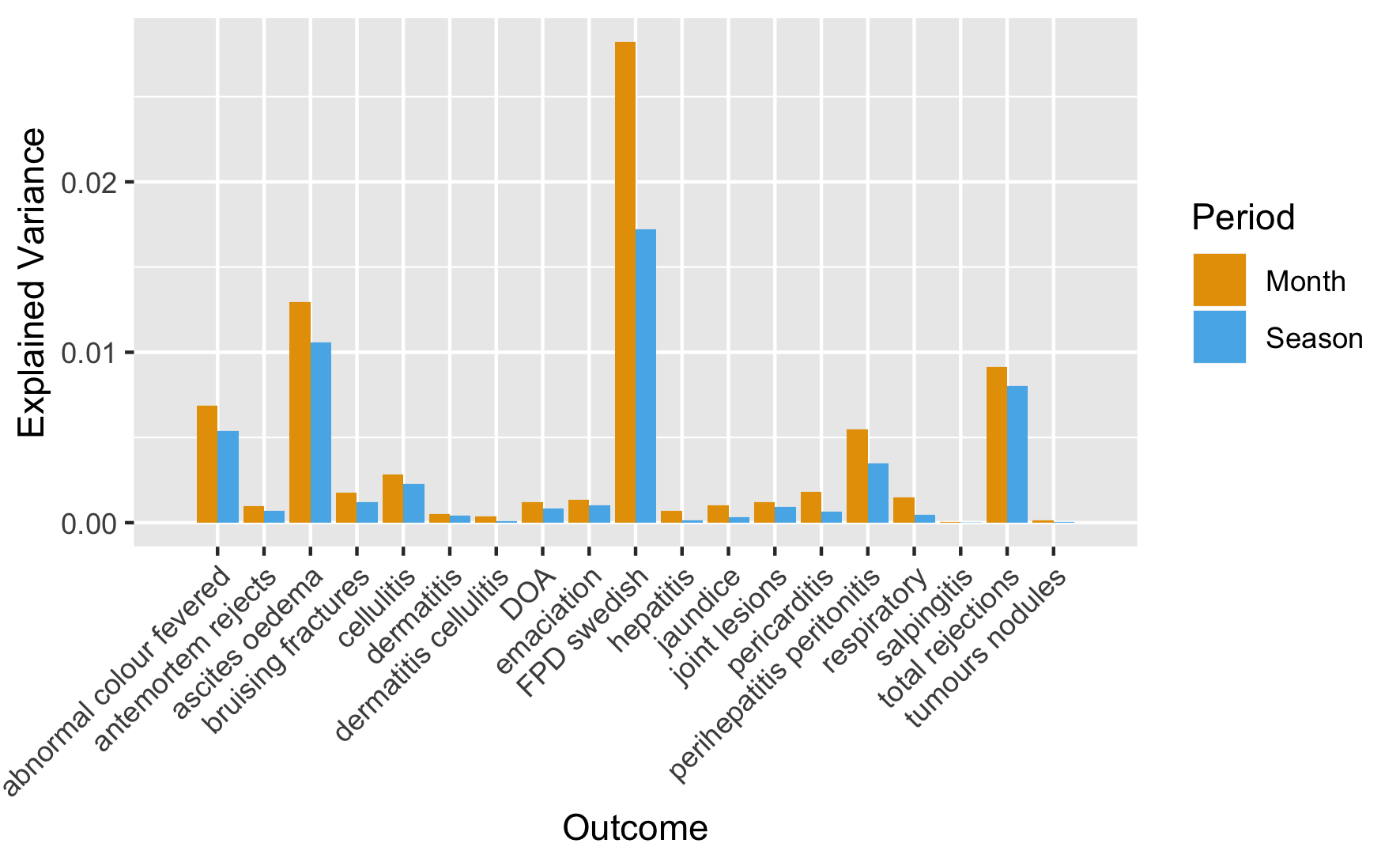


Figure S18. Explained Variance of Month and Season in Outcome Variables


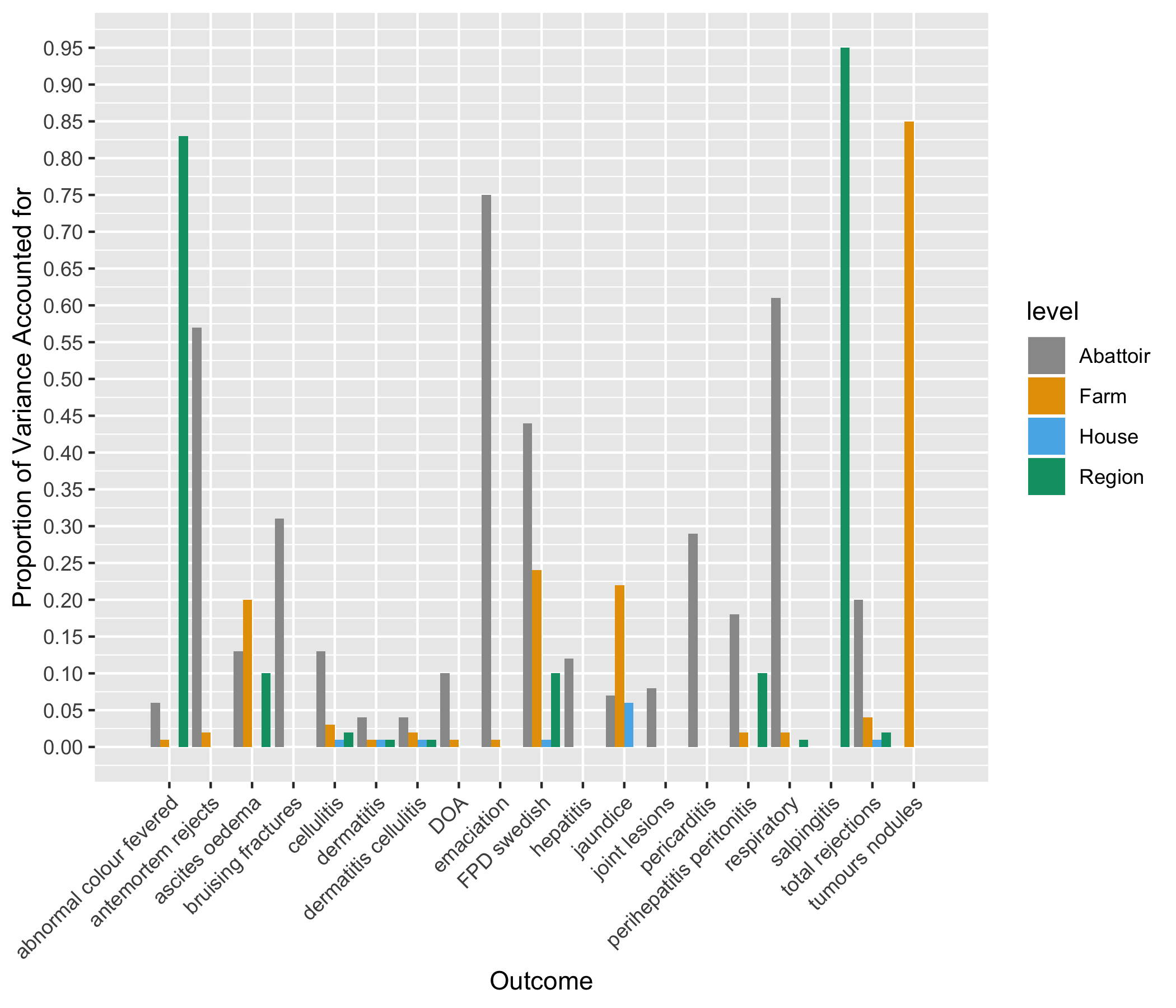


Figure S19. Proportion of Variance Explained in Each Outcome by Grouping Variable

Figures S20 to S39: Risk Factors for outcome measures. Note some measures were only recorded in one time period or another (2010-2014 or 2016-2018) and that there is no data for any measure between 9^th^ June 2014 and 1^st^ April 2016.


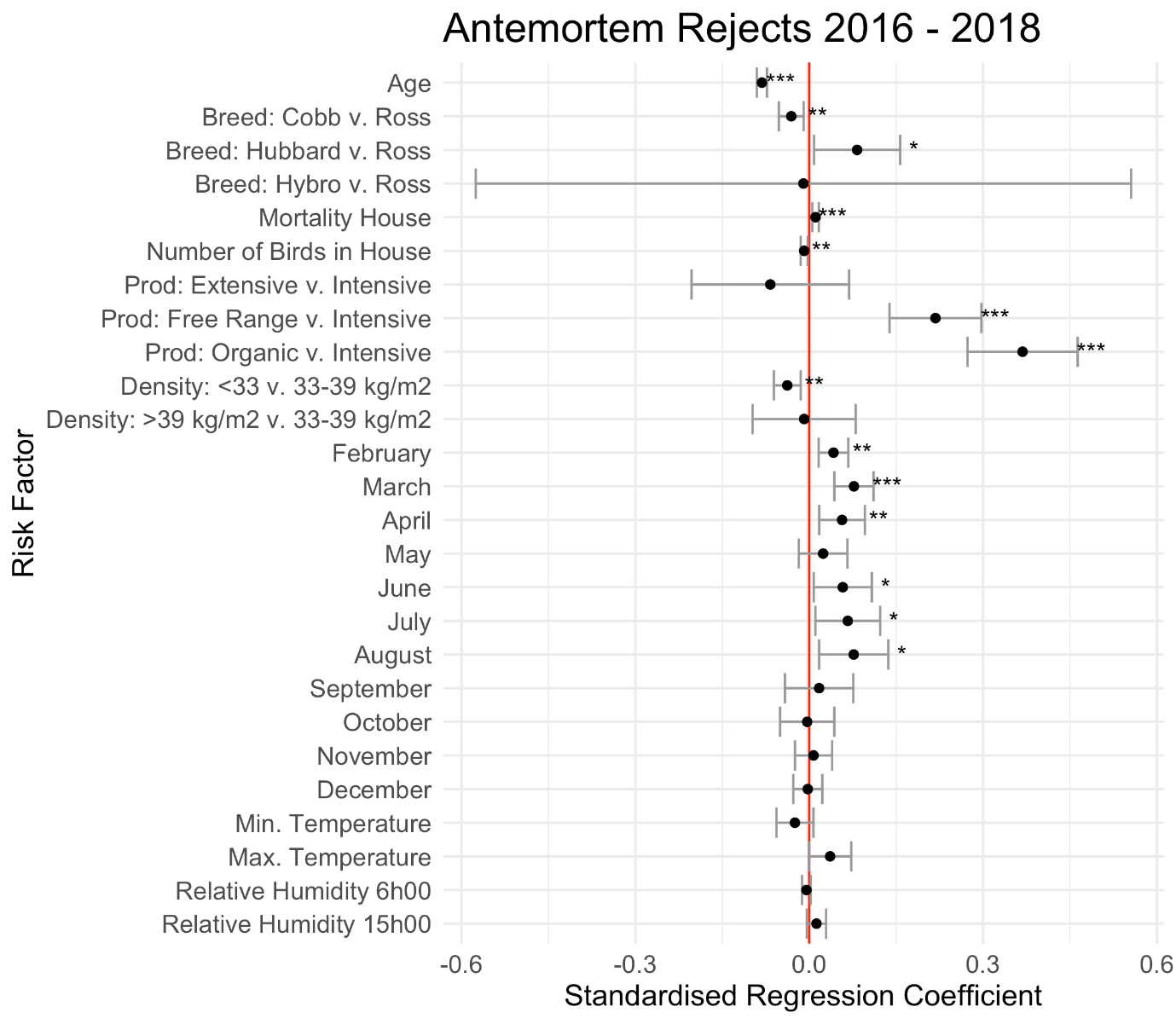


Figure S20. Risk Factors for Antemortem Rejects in the 2016-2018 dataset. Confidence intervals are 95%. ***p<0.001, **p<0.01, *p<0.05.


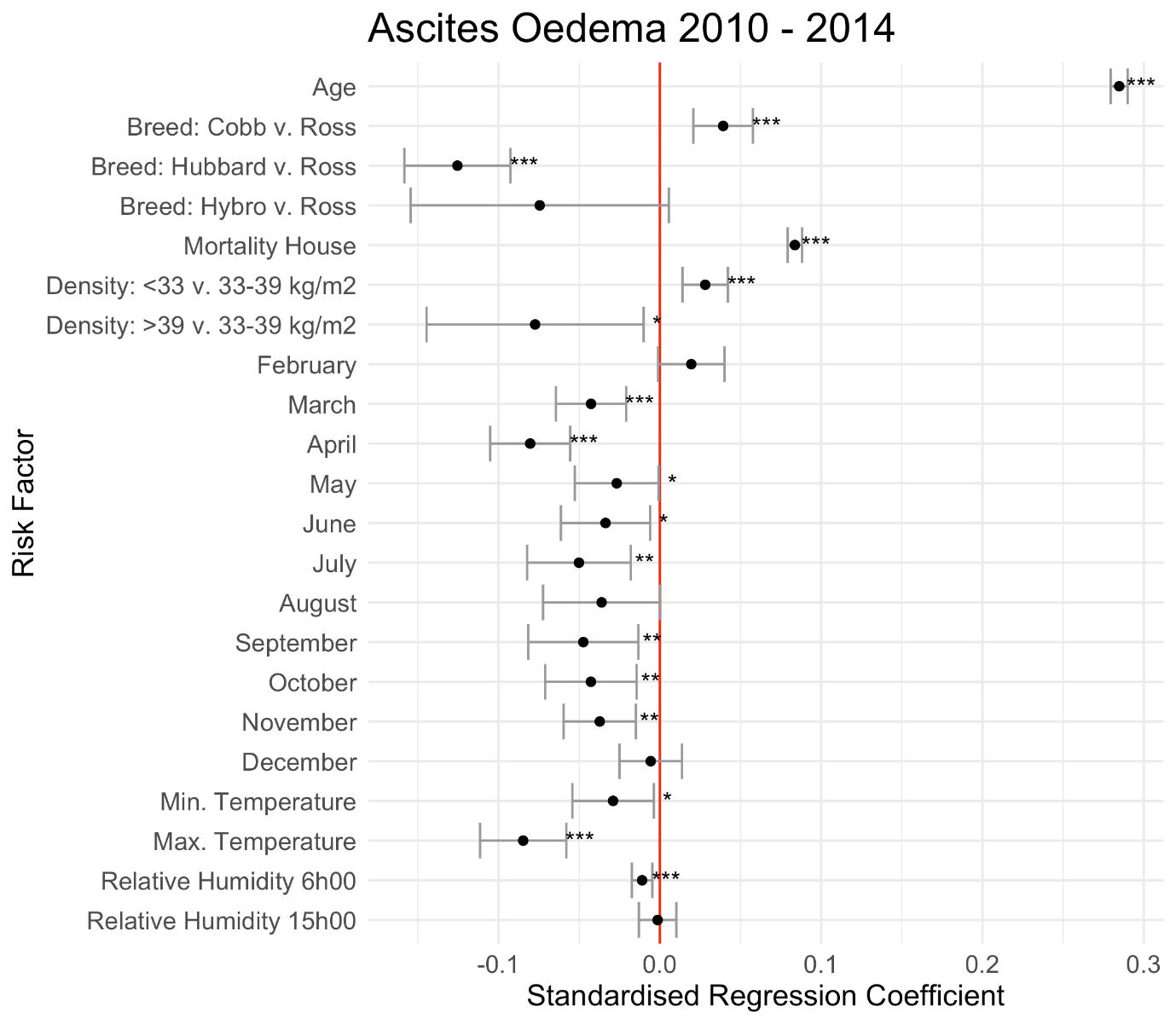


Figure S21. Risk Factors for Ascites/Oedema in the 2010-2014 dataset. Confidence intervals are 95%. ***p<0.001, **p<0.01, *p<0.05.


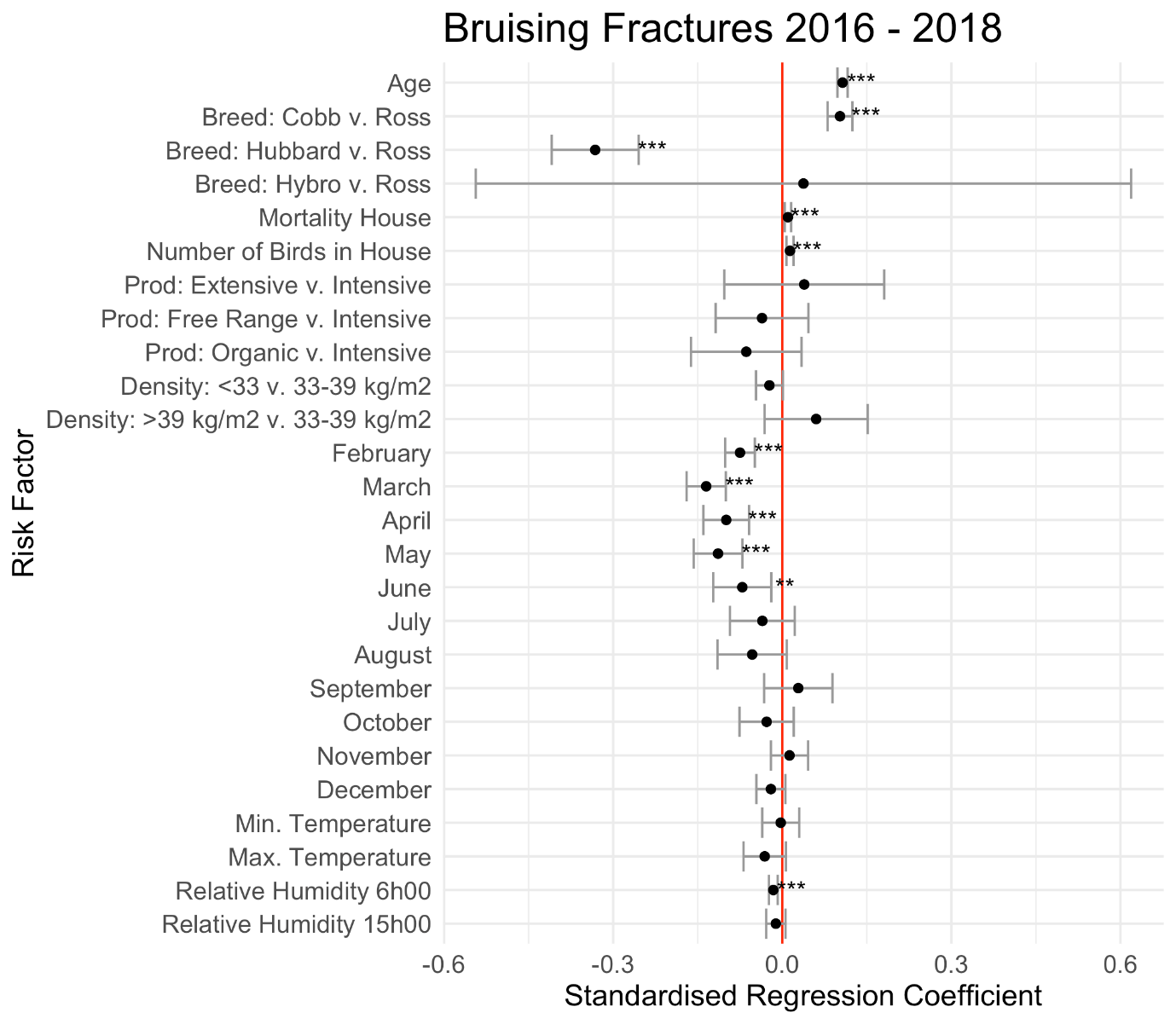


Figure S22. Risk Factors for Bruising Fractures in the 2016-2018 dataset. Confidence intervals are 95%. ***p<0.001, **p<0.01, *p<0.05.
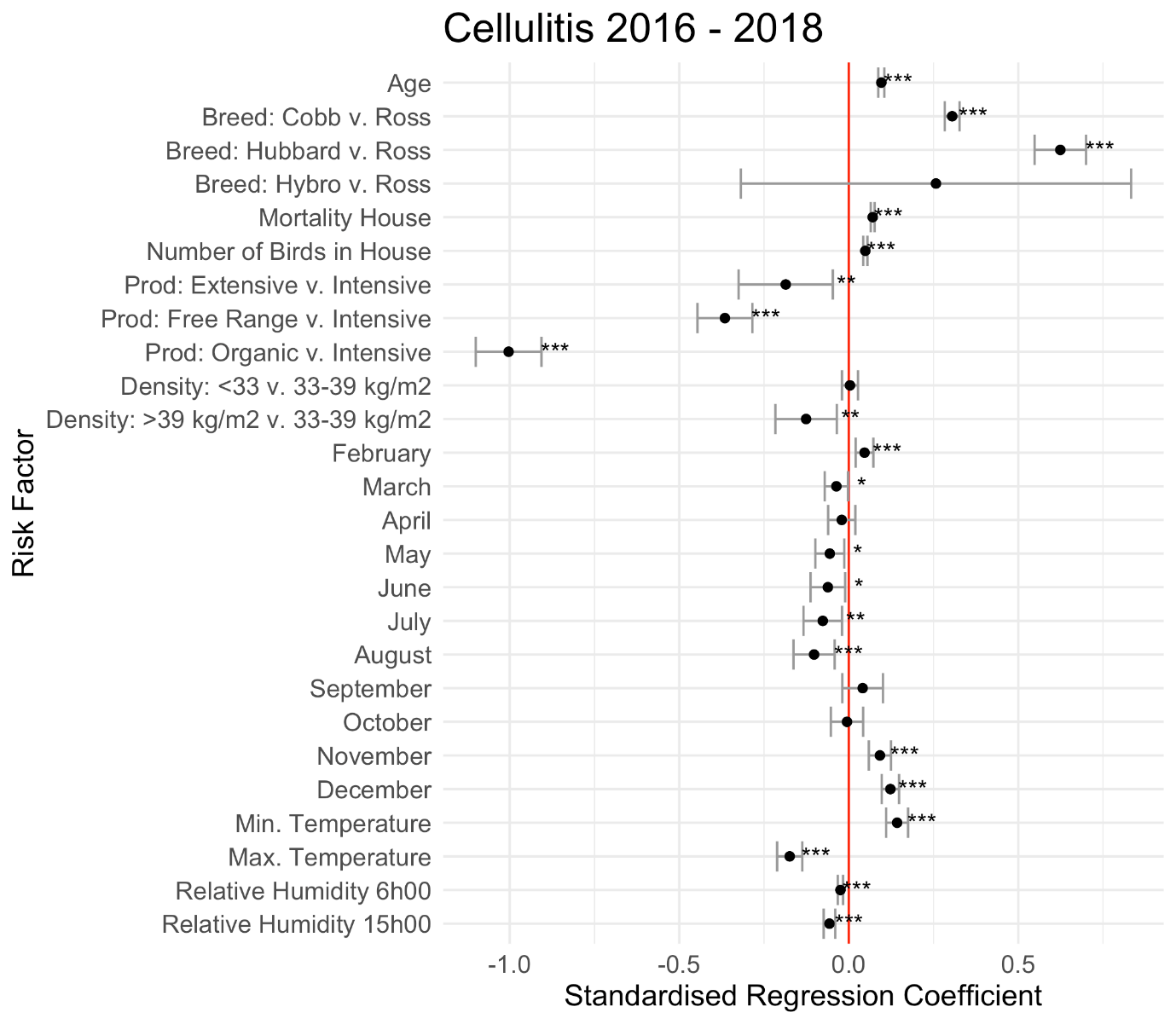


Figure S23. Risk Factors for Cellulitis in the 2016-2018 dataset. Confidence intervals are 95%. ***p<0.001, **p<0.01, *p<0.05.
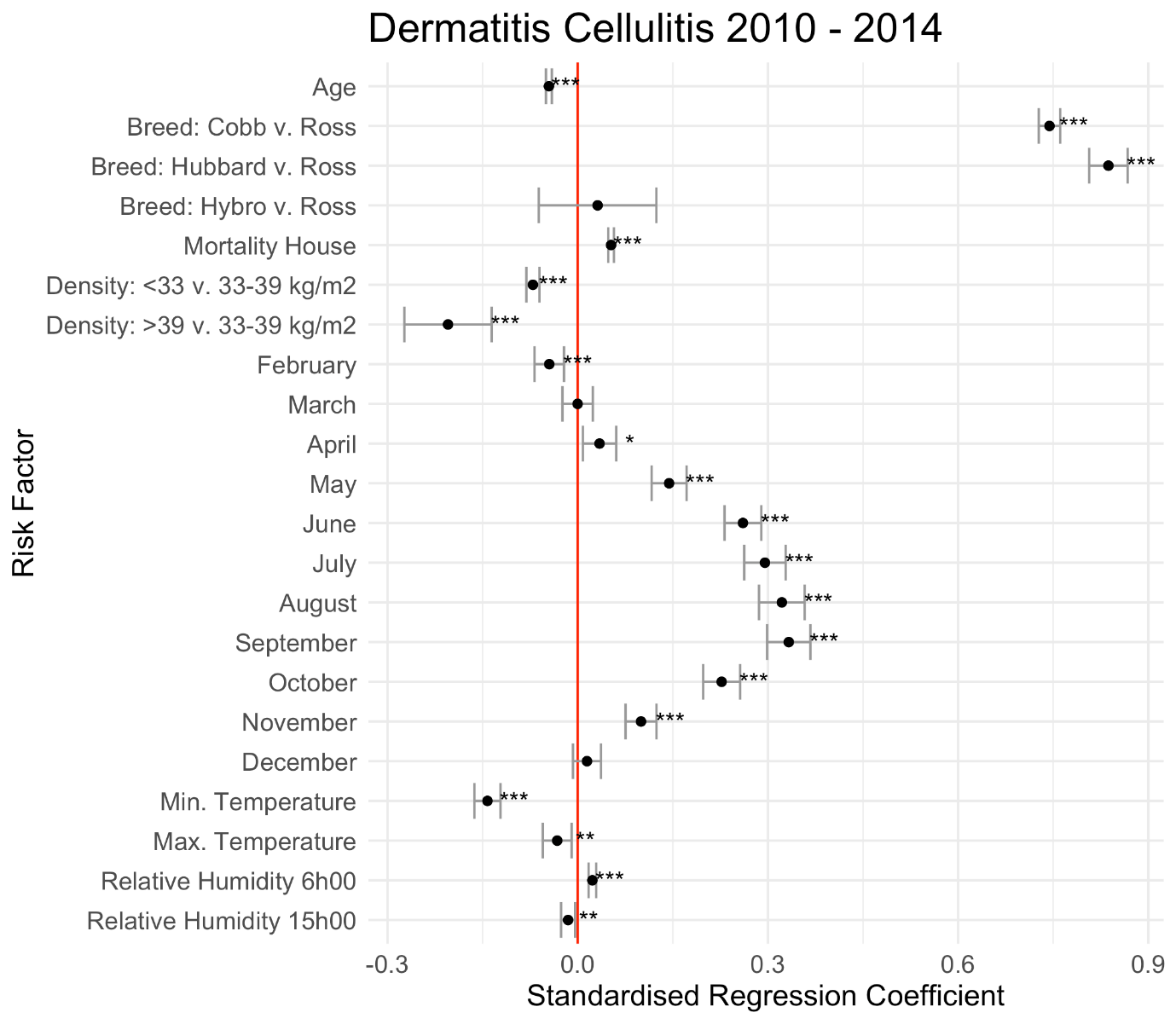


Figure S24. Risk Factors for Dermatitis Cellulitis in the 2010-2014 dataset. Confidence intervals are 95%. ***p<0.001, **p<0.01, *p<0.05.
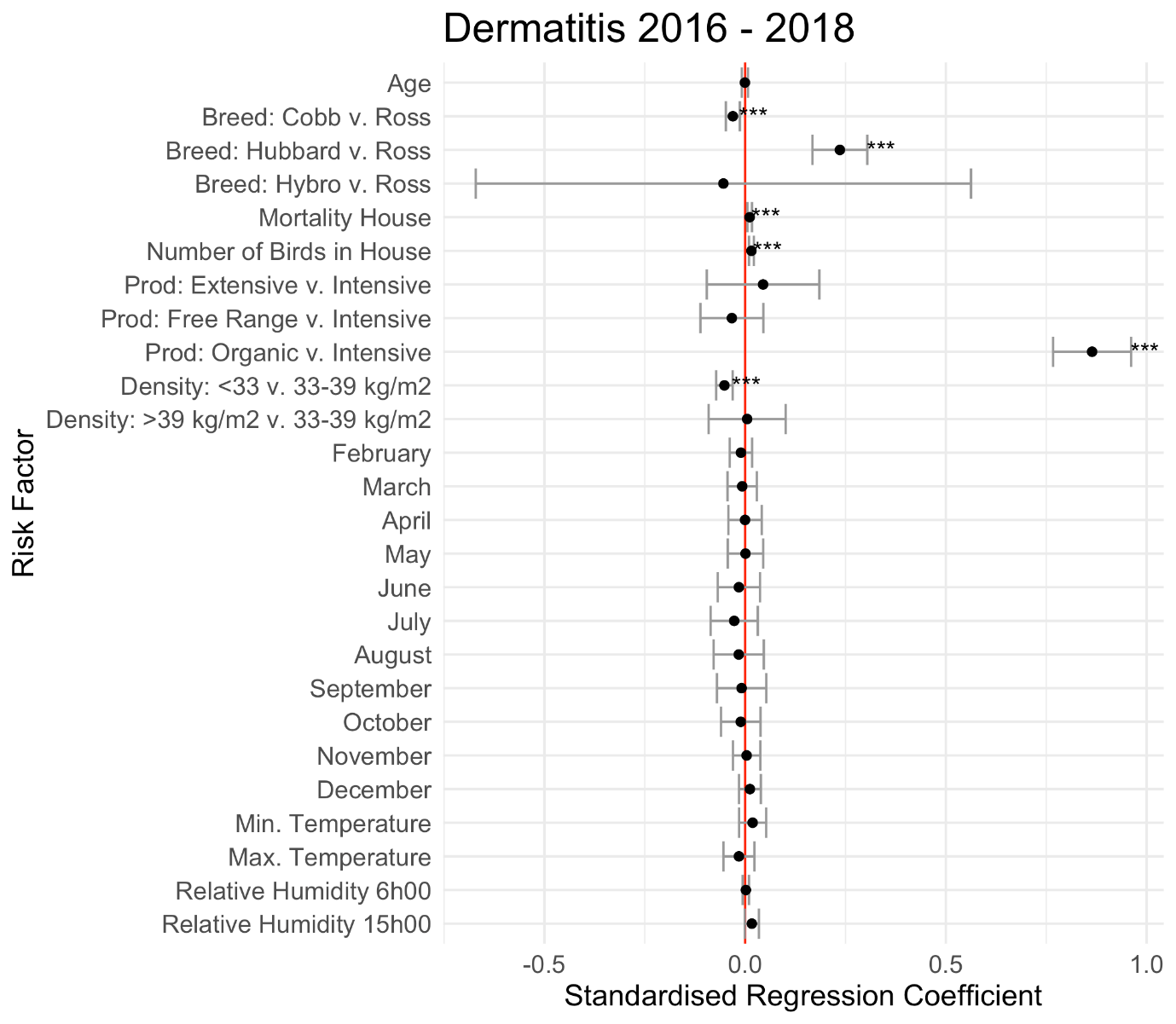


Figure S25. Risk Factors for Dermatitis in the 2016-2018 dataset. Confidence intervals are 95%. ***p<0.001, **p<0.01, *p<0.05.
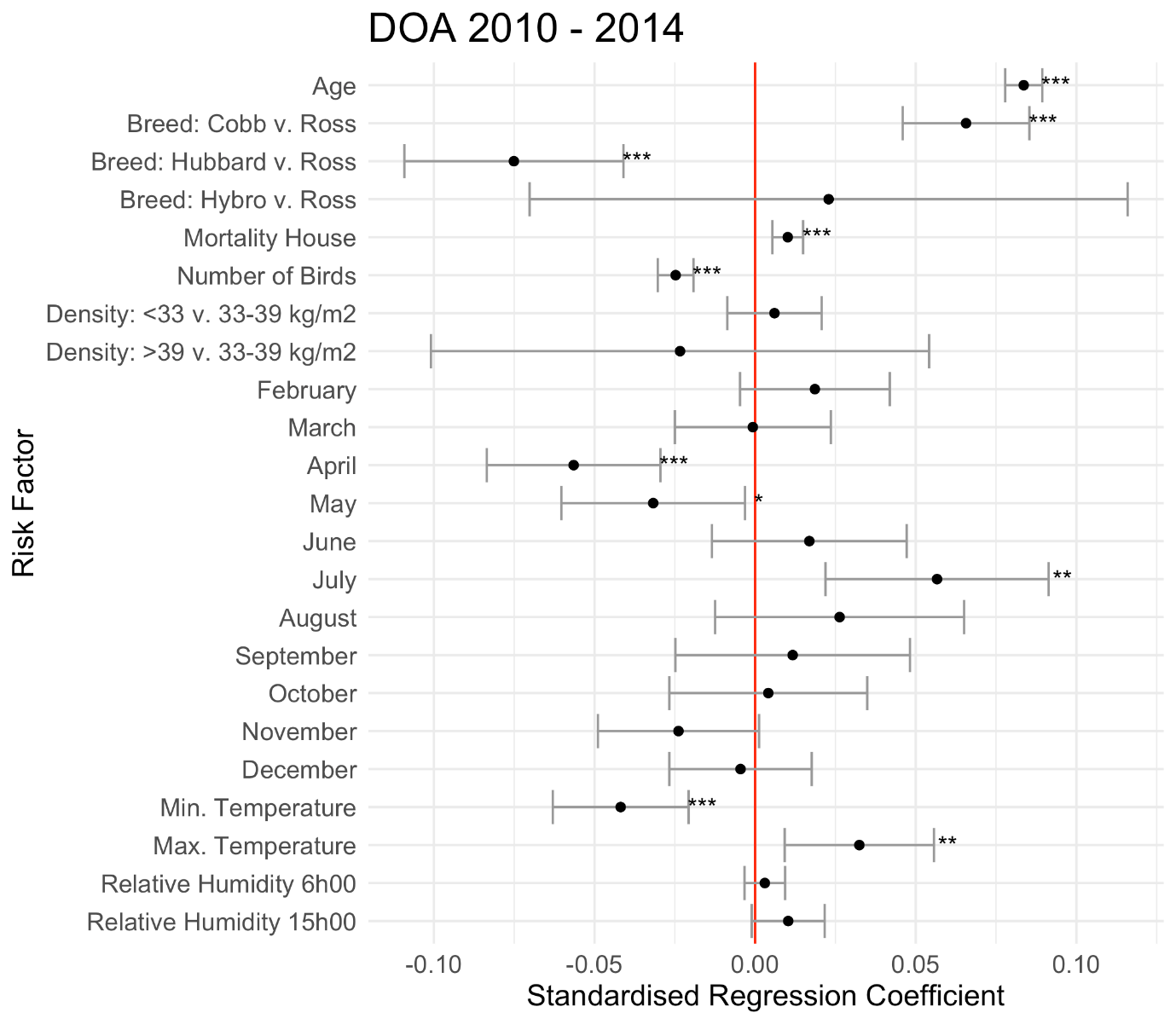


Figure S26. Risk Factors for Dead on Arrival (DOA) in the 2010-2014 dataset. Confidence intervals are 95%. ***p<0.001, **p<0.01, *p<0.05.
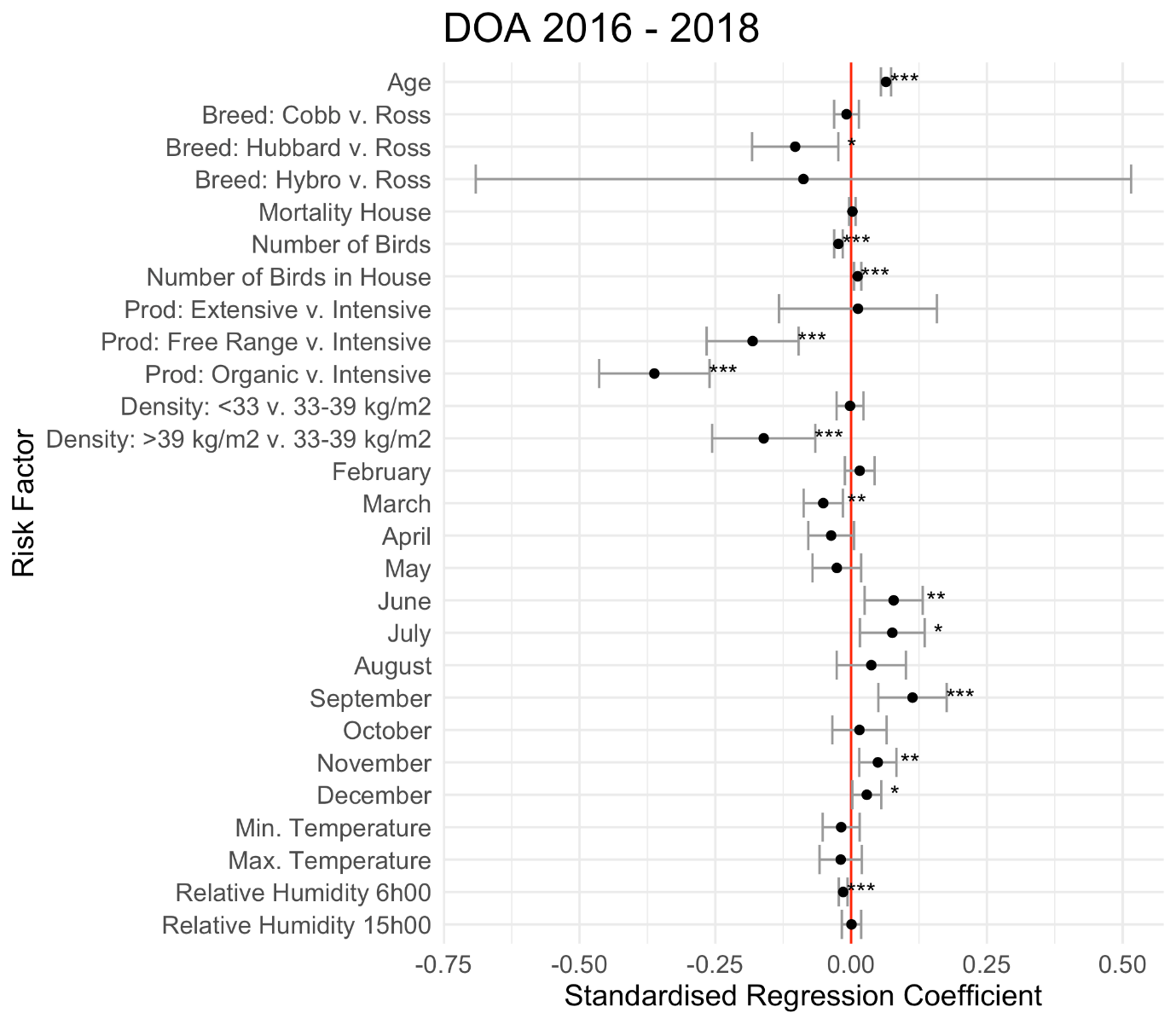


Figure S27. Risk Factors for Dead on Arrival (DOA) in the 2016-2018 dataset. Confidence intervals are 95%. ***p<0.001, **p<0.01, *p<0.05.
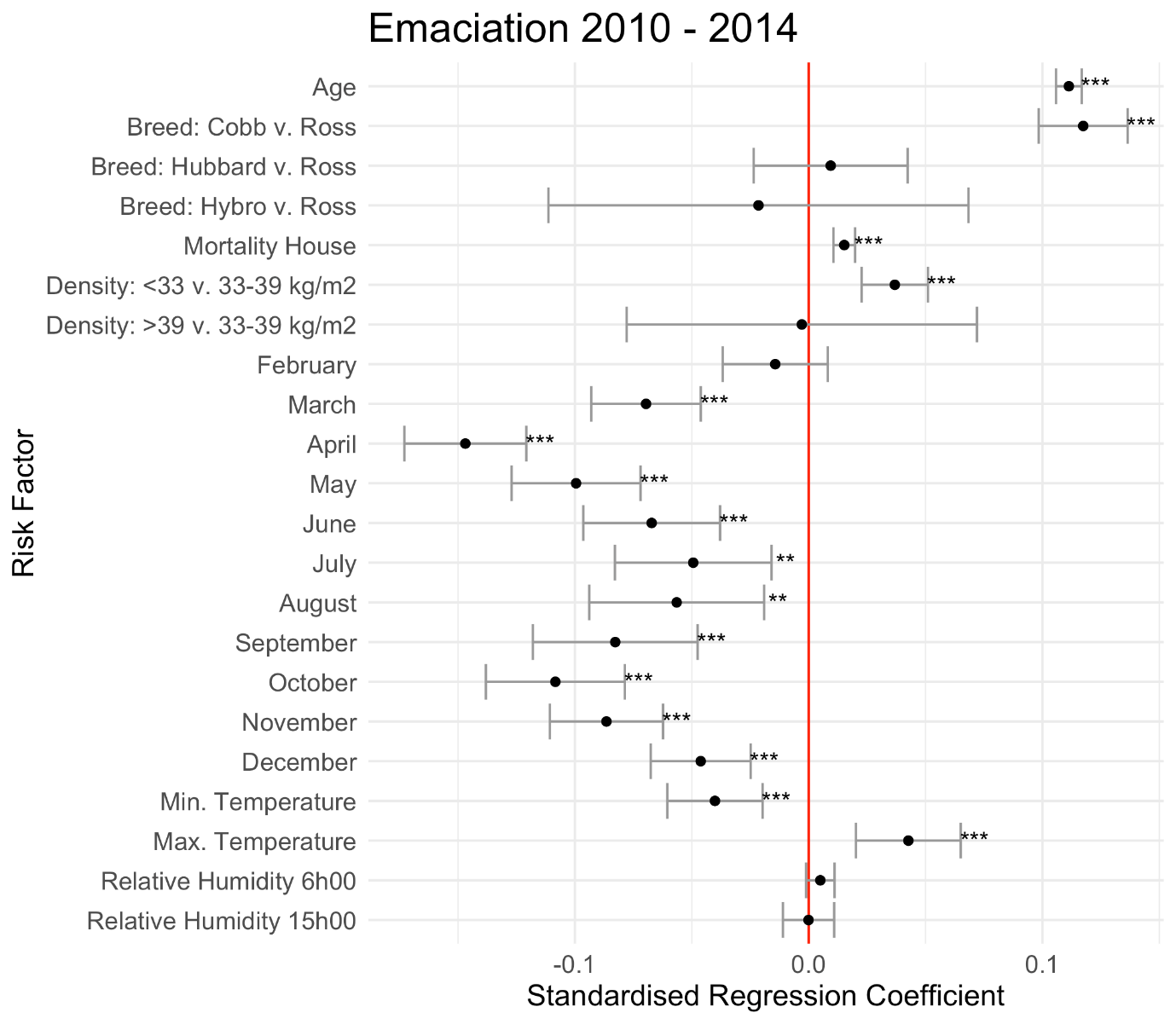


Figure S28. Risk Factors for Emaciation in the 2010-2014 dataset. Confidence intervals are 95%. ***p<0.001, **p<0.01, *p<0.05.
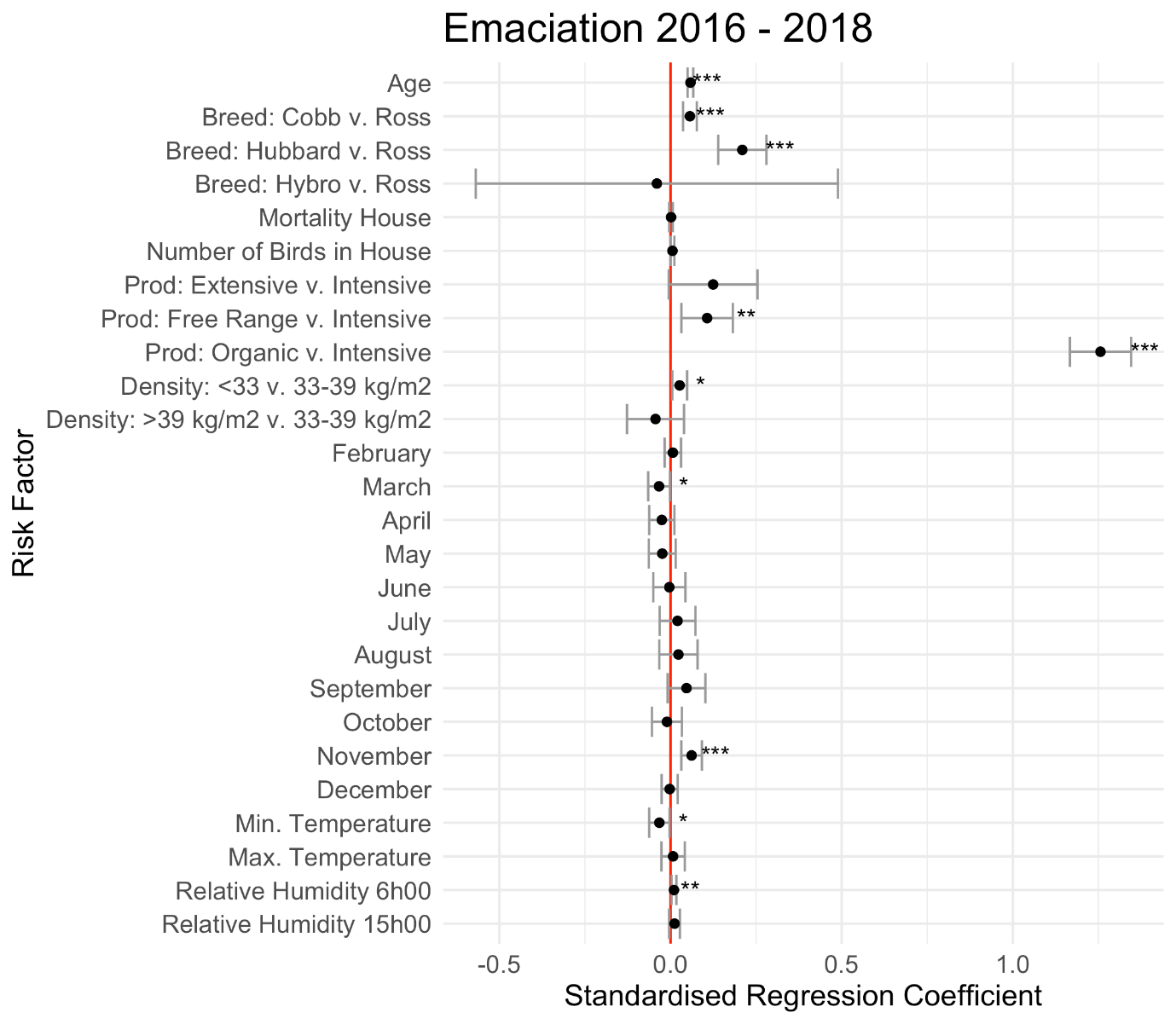


Figure S29. Risk Factors for Emaciation in the 2016-2018 dataset. Confidence intervals are 95%. ***p<0.001, **p<0.01, *p<0.05.
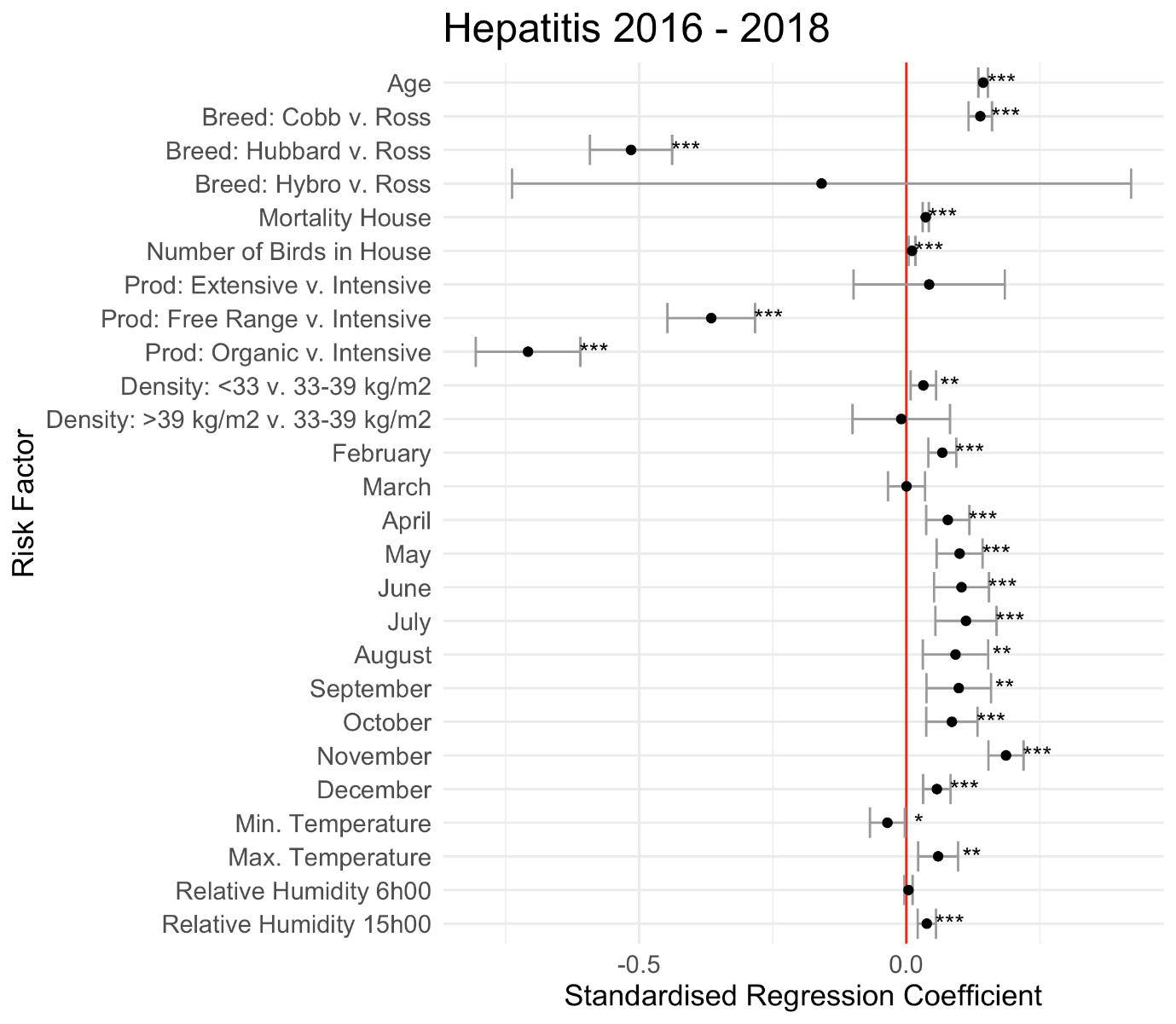


Figure S30. Risk Factors for Hepatitis in the 2016-2018 dataset. Confidence intervals are 95%. ***p<0.001, **p<0.01, *p<0.05.
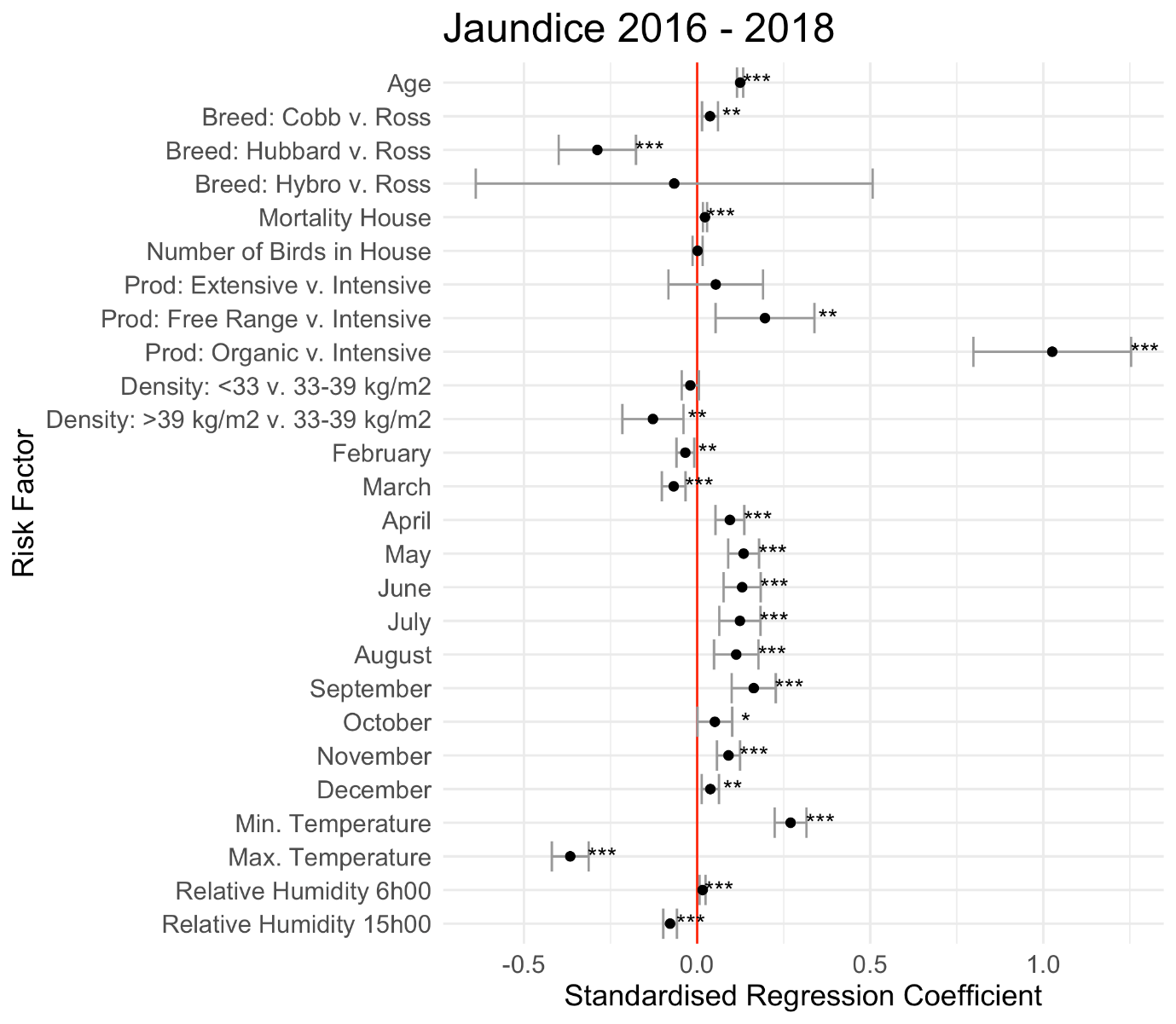


Figure S31. Risk Factors for Jaundice in the 2016-2018 dataset. Confidence intervals are 95%. ***p<0.001, **p<0.01, *p<0.05.
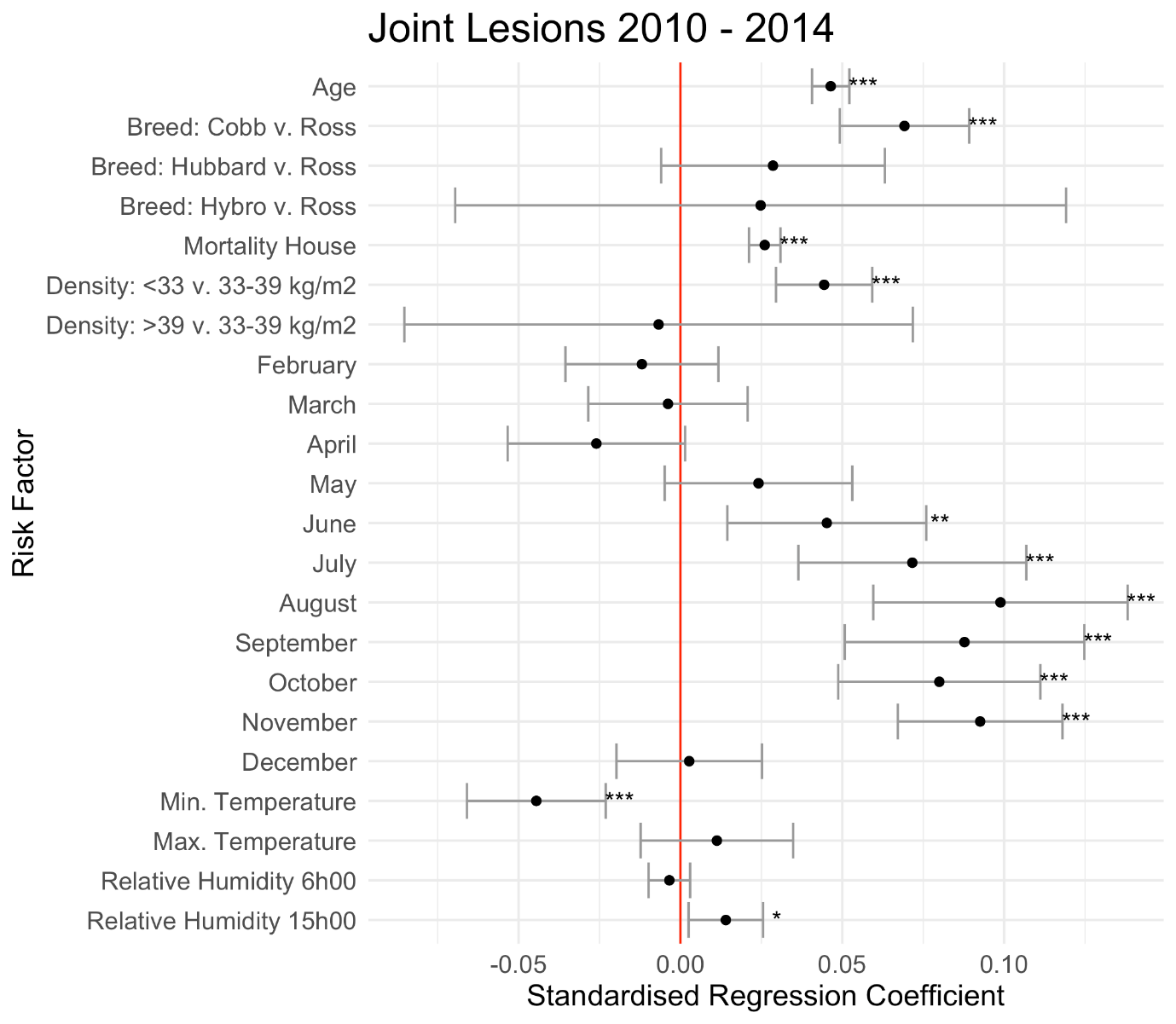


Figure S32. Risk Factors for Joint Lesions in the 2010-2014 dataset. Confidence intervals are 95%. ***p<0.001, **p<0.01, *p<0.05.
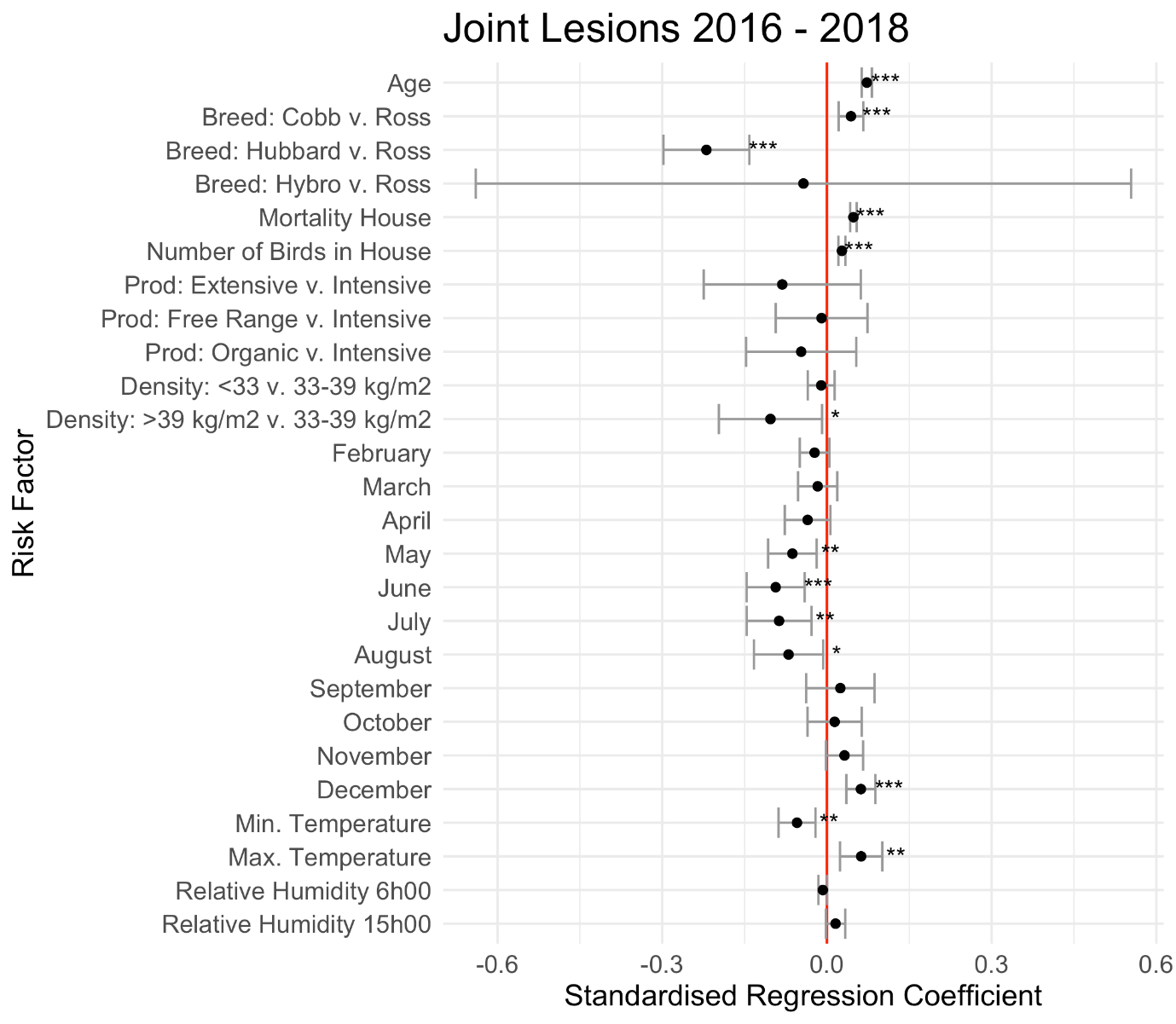


Figure S33. Risk Factors for Joint Lesions in the 2016-2018 dataset. Confidence intervals are 95%. ***p<0.001, **p<0.01, *p<0.05.
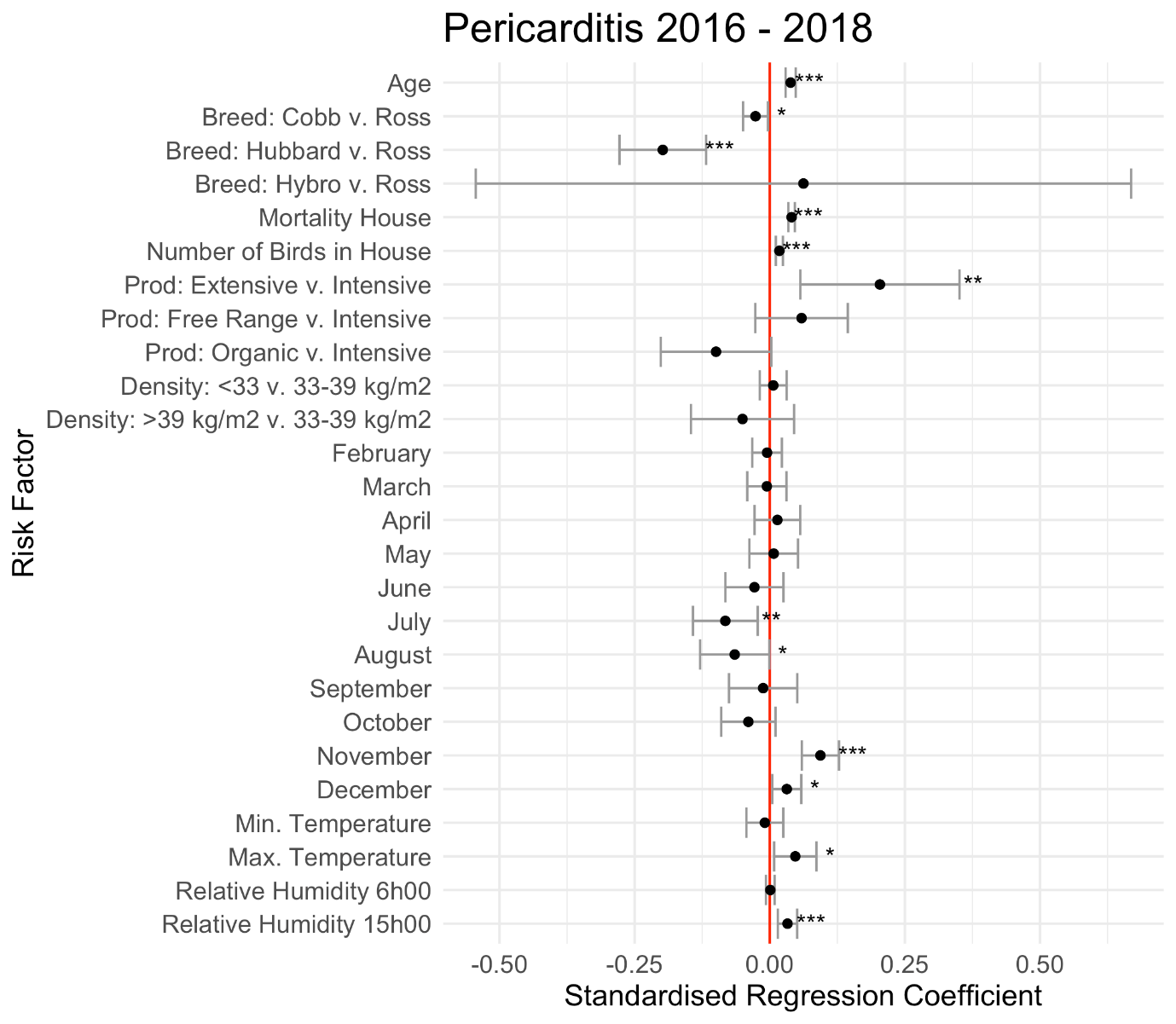


Figure S34. Risk Factors for Pericarditis in the 2016-2018 dataset. Confidence intervals are 95%. ***p<0.001, **p<0.01, *p<0.05.
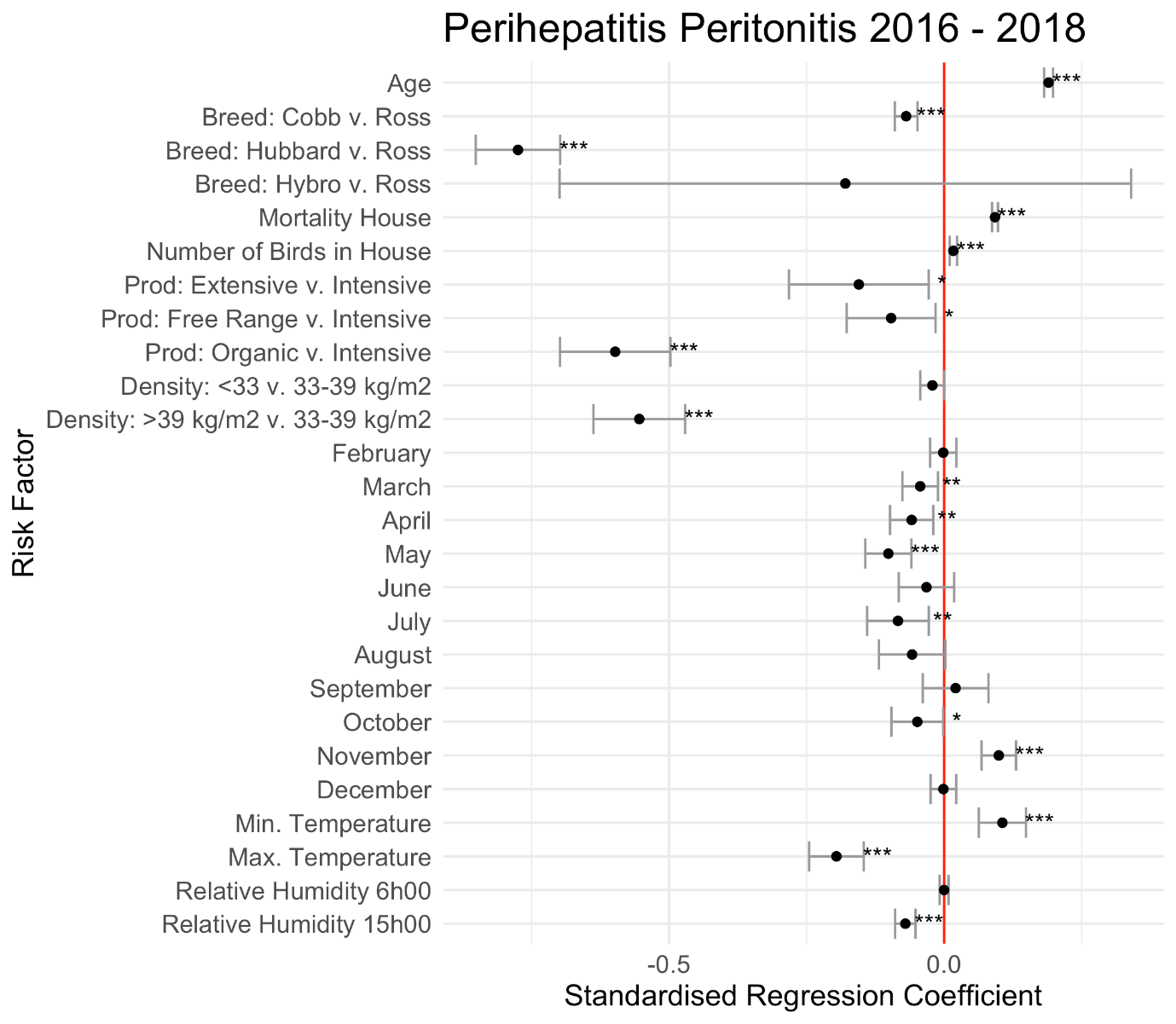


Figure S35. Risk Factors for Perihepatitis/ Pericarditis in the 2016-2018 dataset. Confidence intervals are 95%. ***p<0.001, **p<0.01, *p<0.05.
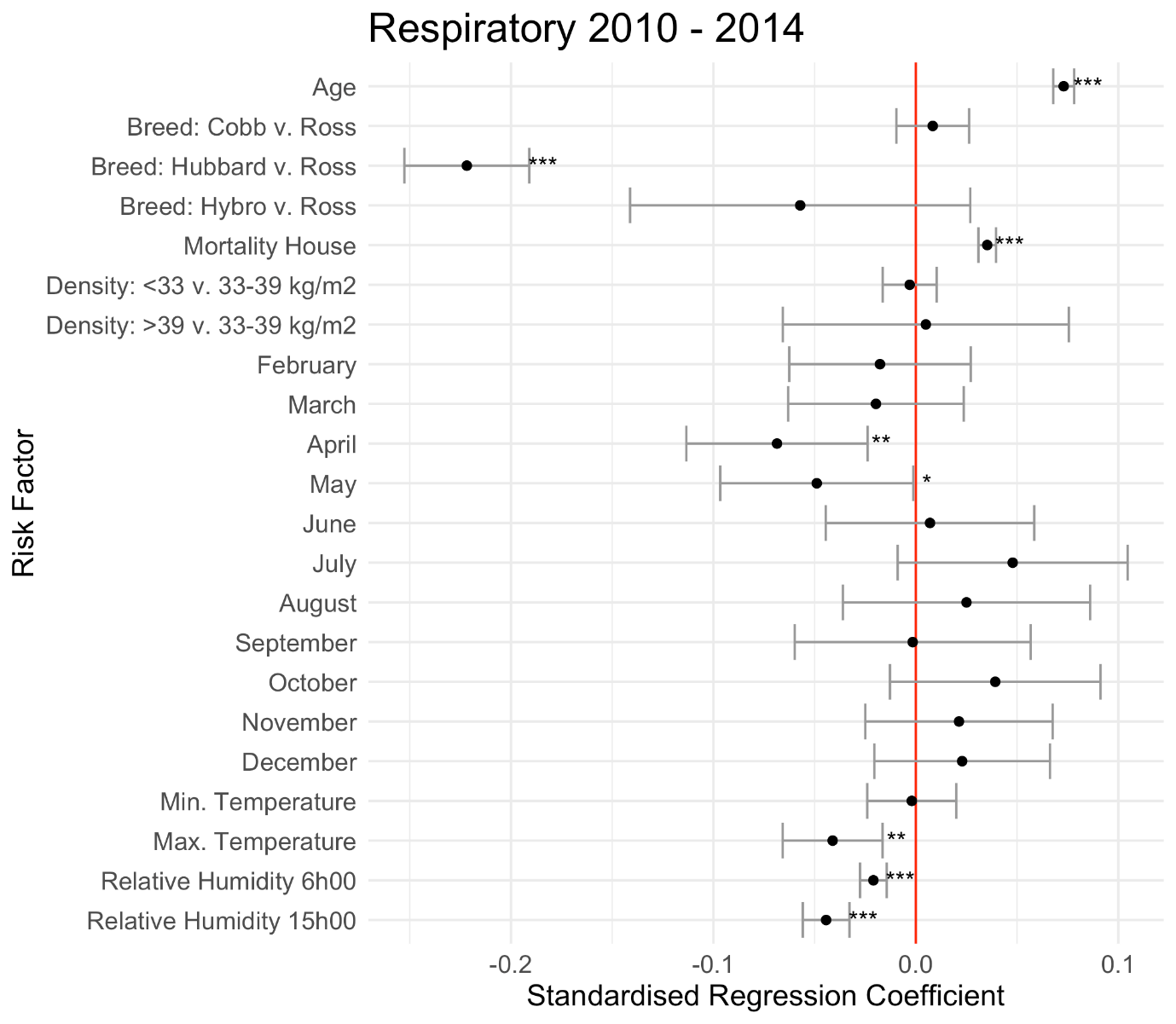


Figure S36. Risk Factors for Respiratory disease in the 2010-2014 dataset. Confidence intervals are 95%. ***p<0.001, **p<0.01, *p<0.05.
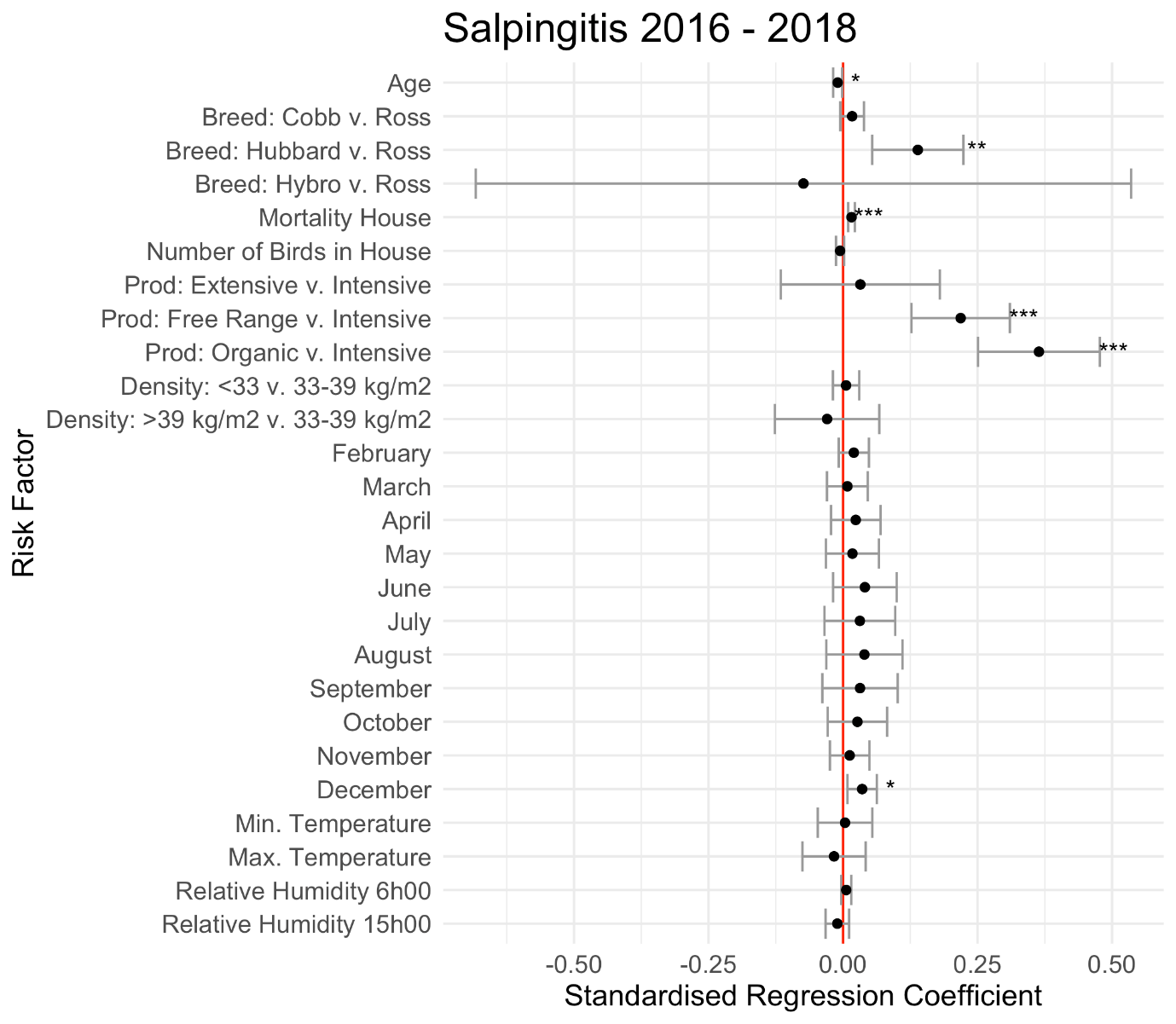


Figure S37. Risk Factors for Salpingitis in the 2016-2018 dataset. Confidence intervals are 95%. ***p<0.001, **p<0.01, *p<0.05.
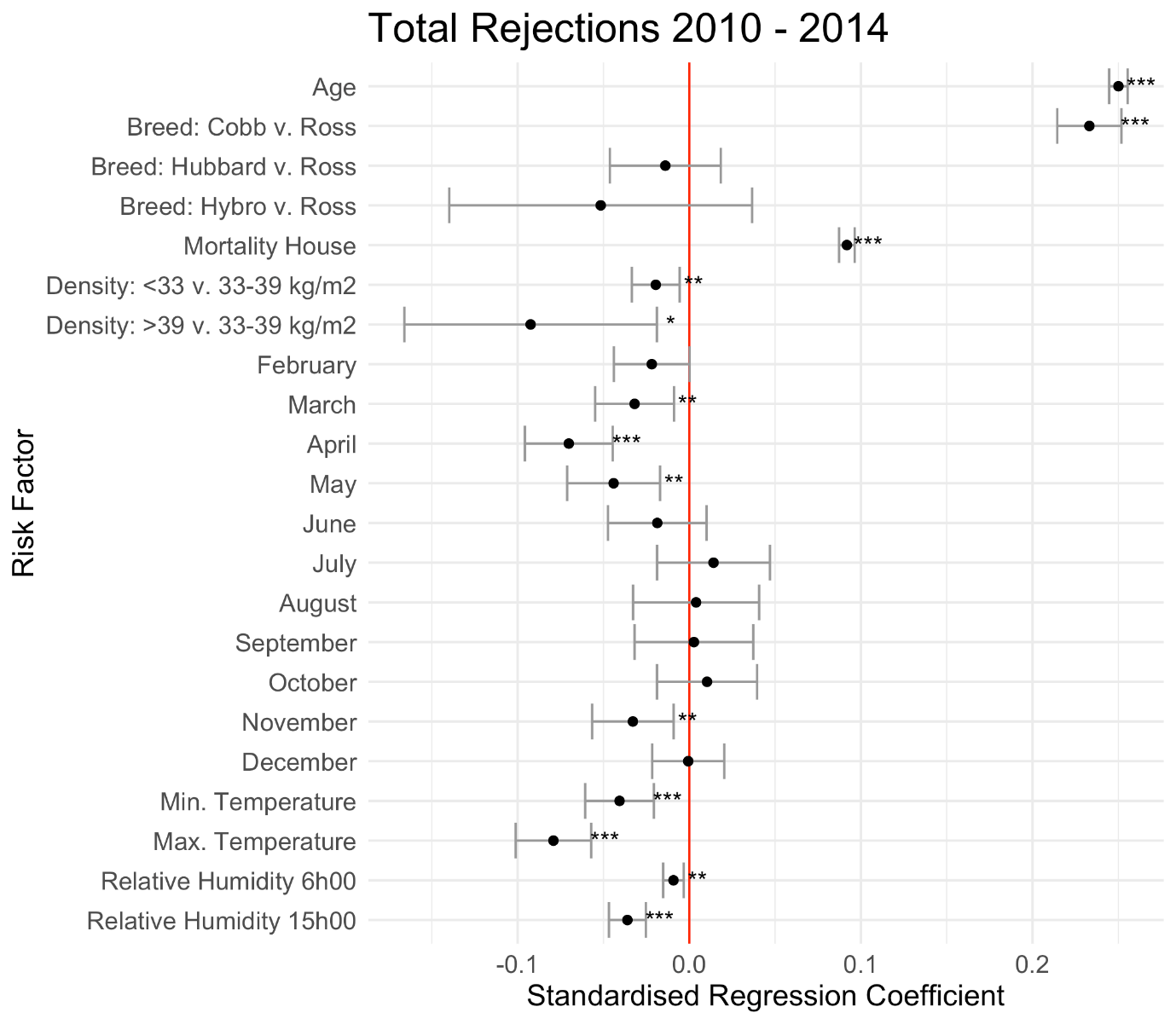


Figure S38. Risk Factors for Total Rejections in the 2010-2014 dataset. Confidence intervals are 95%. ***p<0.001, **p<0.01, *p<0.05.
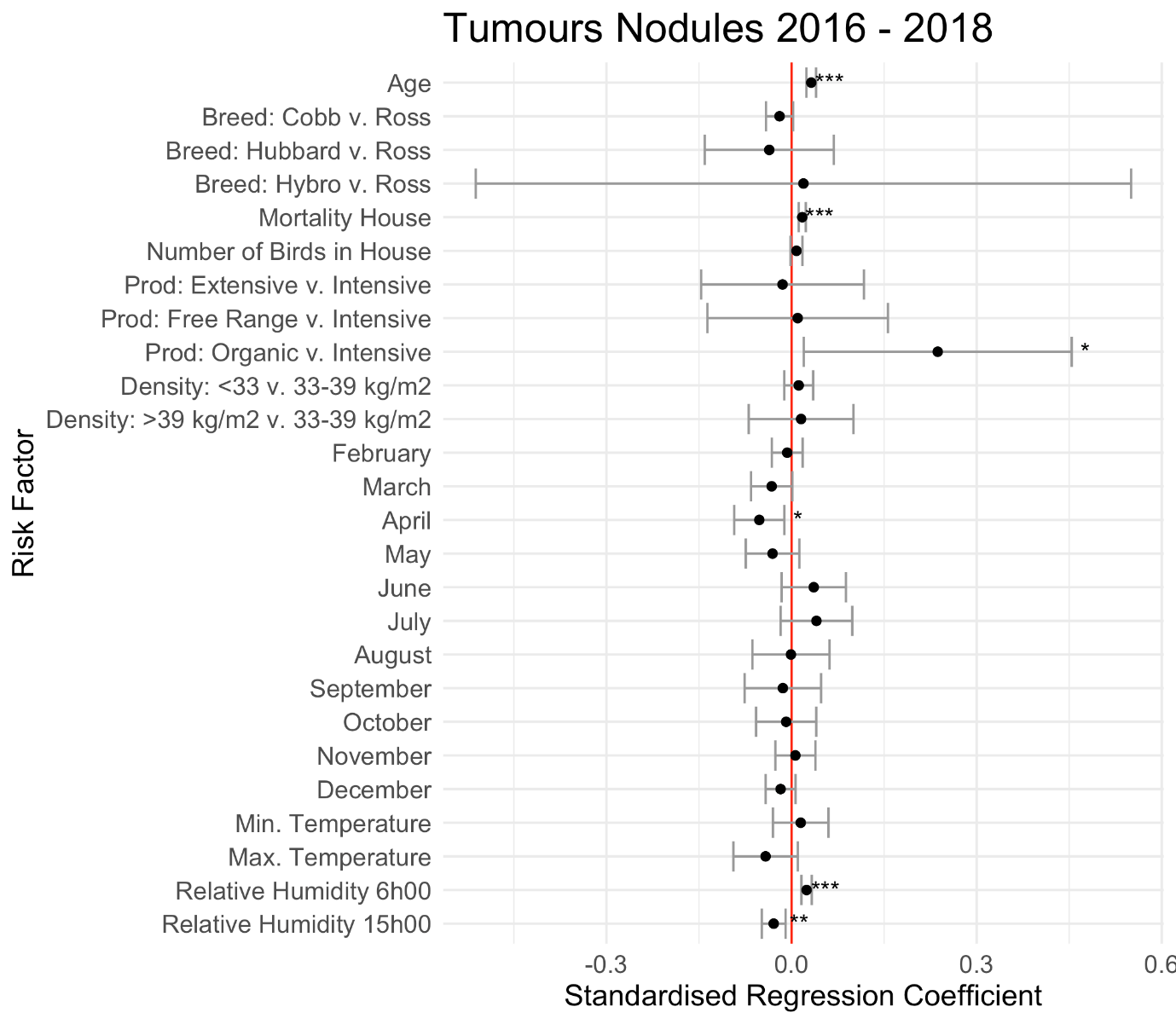


Figure S39. Risk Factors for Tumours/ Nodules in the 2016-2018 dataset. Confidence intervals are 95%. ***p<0.001, **p<0.01, *p<0.05.
